# Ion association with tetra-*n*-alkylammonium cations stabilizes higher-oxidation-state neptunium dioxocations

**DOI:** 10.1038/s41467-018-07982-5

**Published:** 2019-01-04

**Authors:** Shanna L. Estes, Baofu Qiao, Geng Bang Jin

**Affiliations:** 1 Chemical Sciences and Engineering Division, Argonne National Laboratory, 9700 S. Cass Ave., Argonne, IL 60439 USA; 2 Environmental Engineering and Earth Sciences, Clemson University, 342 Computer Court, Anderson, SC 29625 USA; 3 Department of Materials Science and Engineering, Northwestern University, Evanston, IL 60208 USA; 4 3M Corporate Research Analytical Laboratory, 3M Center, Bldg. 201-BS-03, St. Paul, MN 55144 USA

## Abstract

Extended-coordination sphere interactions between dissolved metals and other ions, including electrolyte cations, are not known to perturb the electrochemical behavior of metal cations in water. Herein, we report the stabilization of higher-oxidation-state Np dioxocations in aqueous chloride solutions by hydrophobic tetra-*n*-alkylammonium (TAA^+^) cations—an effect not exerted by fully hydrated Li^+^ cations under similar conditions. Experimental and molecular dynamics simulation results indicate that TAA^+^ cations not only drive enhanced coordination of anionic Cl^–^ ligands to Np^V/VI^ but also associate with the resulting Np complexes via non-covalent interactions, which together decrease the electrode potential of the Np^VI^/Np^V^ couple by up to 220 mV (ΔΔ*G* = −22.2 kJ mol^−1^). Understanding the solvation-dependent interplay between electrolyte cations and metal–oxo species opens an avenue for controlling the formation and redox properties of metal complexes in solution. It also provides valuable mechanistic insights into actinide separation processes that widely use quaternary ammonium cations as extractants or in room temperature ionic liquids.

## Abstract (web summary)

The electrochemical behaviour of redox-active metal cations foremost depends on the metal centre’s inner-sphere coordination environment. Here the authors show that electrolyte cations unexpectedly stabilize higher-oxidation-state neptunium dioxocations in water through extended-coordination sphere interactions.

## Introduction

The electrochemical behavior of redox-active metal cations is inherently dependent on the inner-sphere coordination environment surrounding the metal center^1,2^. Although this fundamental principle holds true, there is increasing evidence that the chemical environment beyond the first coordination sphere can also influence the observed electrochemical properties^3–7^. For example, by measuring electrode potentials in organic media and by characterizing solid reaction products, several recent studies have demonstrated that direct second-sphere coordination of strong Lewis acids to oxo groups enhances the redox activity of metal–oxo complexes, including increasing the oxidizing capacity of manganese–oxo clusters in the oxygen-evolving complex of photosystem II^5,6^ and facilitating oxo group functionalization and reduction of the uranyl ion, UO_2_^2+^ ^3,4^. For aqueous systems, however, the hydration of charged species (e.g., ion–solvent interactions) is expected to inhibit or outcompete any direct ion–ion interactions between molecular metal complexes and other cations^8^, such that the influence of extended-coordination environments on the redox chemistry of metals dissolved in water has been overlooked.

To address this knowledge gap, we examined the redox behavior of Np^V^ in acidic aqueous chloride solutions as a function of electrolyte cation. The chemical properties of Np^V^ make it an excellent choice for studying the influence of extended-coordination environments on the redox chemistry of aqueous metal ions. First, in aqueous solutions, high-valent early actinides exist as dioxo actinyl cations (An^V^O_2_^+^ or An^VI^O_2_^2+^, where An = U, Np, Pu, and Am), which have a unique and stable nearly linear structure, with two strong An–O covalent bonds^9^. The partial negative character on the yl (yl = actinyl) oxygen atoms facilitates interactions with other cations^10,11^, including other actinyl cations^12–16^, which is generally termed cation–cation interaction (CCI)^12^. This property, namely the reactivity of the actinyl oxo group, provides a chemical path for defined second-coordination sphere interactions that do not perturb the inner-coordination sphere (the actinyl unit). Compared to An^VI^O_2_^2+^ complexes, the lower nuclear charge of the An^V^ increases the Lewis basicity of the yl–oxo in An^V^O_2_^+^ complexes, enhancing extended-coordination sphere interactions for An^V^ cations^17^. Additionally, of the An^V^O_2_^+^ species, Np^V^O_2_^+^ is the most thermodynamically stable^18^. Second, unlike transition-metal–oxo complexes that readily undergo hydrolysis and oligomerization, of which the latter can compete with second-coordination sphere bonding, hydrolysis and oligomerization of Np^V^O_2_^+^ are unlikely under mildly acidic conditions^19^. In fact, previous studies, including EXAFS, indicate that Np^V^ speciation in acidic chloride solutions (pH = 2, [Cl^–^] ≤ 6 M) is dominated by a simple monomeric pentahydrate complex, [Np^V^O_2_(H_2_O)_5_]^+^ ^20^. Third, in aqueous solutions, the Np^VI^/Np^V^ redox couple is easily accessible (*E*° = +0.959(4) V vs. Ag/AgCl)^18^ and generally exhibits reversible or quasi-reversible electron-transfer properties^21,22^, permitting a quantitative or semiquantitative evaluation of the Np^VI^/Np^V^ electron-transfer thermodynamics.

In the present work, we combine electroanalytical chemistry, vibrational and electronic spectroscopies, X-ray crystallography, and molecular dynamics simulations to characterize the interactions that electrolyte cations (A^+ ^= Li^+^ or tetra-*n*-alkylammonium cations (TAA^+ ^= [NMe_4_]^+^, [NEt_4_]^+^)) have with Np^V^O_2_^+^ cations in aqueous systems, and to understand the effect these interactions have on Np^V^ redox behavior, both in solution and during solution evaporation and crystal formation. The two electrolyte cations, Li^+^ and TAA^+^, which are widely used as non-reacting, charge-balancing ions, have significantly different ionic radii and hydration and H-bonding properties, allowing us to investigate how these properties influence interactions between the electrolyte and neptunyl cations in aqueous solution. Here, we show that hydrophobic TAA^+^ cations unexpectedly stabilize higher-oxidation-state metal–oxo cations dissolved in aqueous solutions, whereas the strong Lewis acid, Li^+^, has no effect on the observed redox behavior. Furthermore, we show that TAA^+^ cations, which are not Lewis acids, influence the redox behavior of metal–oxo cations via non-covalent ion association with the metal–oxo complex, not via direct bonding with the oxo group. These findings highlight the underappreciated influence of electrolyte cations on the redox chemistry of metal cations and underscore the important role that ion–solvent interactions play in governing extended-coordination sphere interactions between metal cations and other ions in solution.

## Results

### Redox chemistry of Np^V^ in LiCl and NMe_4_Cl solutions

We first probed the redox chemistry of Np^V^ in several acidic aqueous chloride solutions as a function of A^+^ using cyclic voltammetry (CV). In 1 M and 5 M LiCl (pH ≈ 1.3), the oxidation of Np^V^ is quasi-reversible (i.e., controlled by both charge transfer and mass transport), with average half-wave potentials (*E*_1/2_) of +0.924(2) V and +0.929(3) V vs. Ag/AgCl, respectively (Supplementary Discussion, Supplementary Figure 2, and Supplementary Table 1). These electrode potentials are equal (within error), indicating that increases in LiCl concentration and ionic strength do not affect the redox behavior of Np^V^ (Supplementary Figure 3). Furthermore, the electrode potentials measured here for the LiCl solutions agree well with the electrode potentials previously reported for the Np^VI^/Np^V^ couple in 1 M HClO_4_^21^ (Supplementary Table 1). Taken together, these data indicate that the underlying electronic structure and chemical environment of Np^V^ in solutions of 1 or 5 M LiCl and 1 M HClO_4_ are the same, and that Np^V^ remains present predominantly as the free, hydrated cation, [NpO_2_(H_2_O)_5_]^+^, as expected from previous studies^20,23^.

**Fig. 1:**
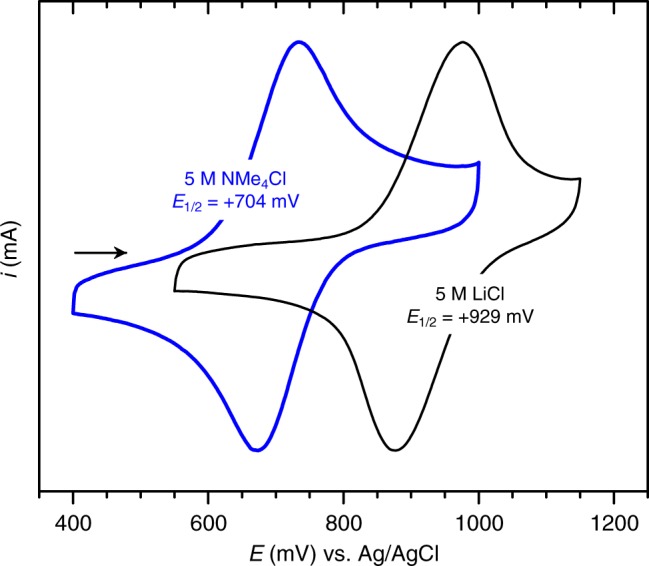
Effect of Li^+^ vs. [NMe_4_]^+^ on the Np^VI^/Np^V^ couple. Current-normalized cyclic voltammetry data for solutions of 5 mM Np^V^ dissolved in 5 M NMe_4_Cl or 5 M LiCl at pH ≈ 1.3. The arrow indicates the initial scan direction. Initial and cathodic switching potential = 400/550 mV; anodic switching potential = 1000/1150 mV; *ν* = 100 mV s^−1^

In contrast, when Li^+^ cations in solution are replaced by TAA^+^ cations, the measured Np^VI^/Np^V^ electrode potential decreases substantially, by as much as 220 mV, indicating that the presence of TAA^+^ cations in solution increases the thermodynamic stability of Np^VI^ relative to Np^V^ (Fig. 1). Specifically, in 1 M, 3 M, and 5 M NMe_4_Cl (pH ≈ 1.3), CV data indicate that electron transfer for the Np^VI^/Np^V^ redox couple is quasi-reversible, with average *E*_1/2_ values of +0.903(4) V, +0.818(7) V, and +0.704(7) V vs. Ag/AgCl, respectively (Supplementary Discussion, Supplementary Figure 4, and Supplementary Table 1). Noting that only the electrolyte cation was changed in these experiments, the large cathodic shifts of the Np^VI^/Np^V^ electrode potential are unexpected, particularly considering that Cl^–^ is a weak ligand^24^. Similar magnitude cathodic shifts of the An^VI^/An^V^ electrode potential in aqueous systems have only been observed in the presence of strong coordinating ligands, such as sulfate^25^, carbonate^26^, or hydroxide^27^. Moreover, the gradual linear decrease of the electrode potential with increasing NMe_4_Cl concentrations provides clear evidence for the formation of one or more additional Np^V^ species^28^, which exhibit greater thermodynamic stabilization toward Np^VI^ and which are in equilibrium with existing [NpO_2_(H_2_O)_5_]^+^ complexes. The Np^VI^/Np^V^ electrode potentials measured in mixed constant-ionic-strength LiCl/NMe_4_Cl solutions and in NEt_4_Cl are similar in magnitude to the electrode potentials measured in NMe_4_Cl solutions with the same TAA^+^ concentration (Supplementary Discussion, Supplementary Figure 5, and Supplementary Table 1), confirming that the shifts of the Np^VI^/Np^V^ electrode potentials are not due to increasing ionic strength and are not specific to solutions containing [NMe_4_]^+^ cations.

### Raman and Vis–NIR absorption spectroscopy

**Fig. 2:**
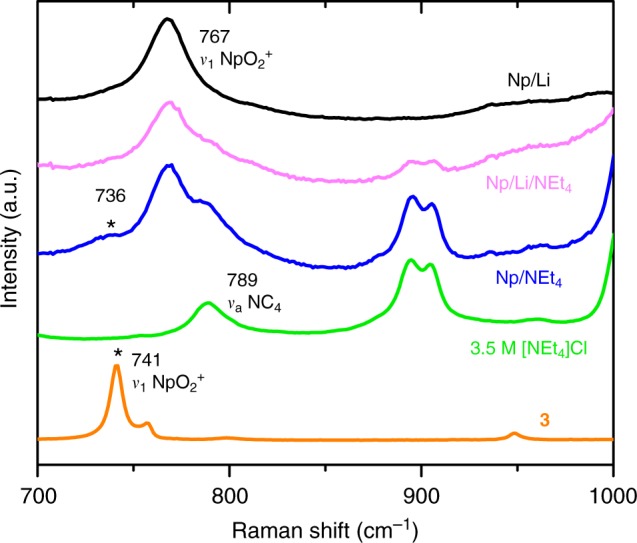
Comparison of Np^V^ Raman spectra. Raman spectra of 0.12 M Np^V^ dissolved in 3.5 M LiCl (Np/Li, black line), 1.75 M LiCl/1.75 M NEt_4_Cl (Np/Li/NEt_4_, magenta line), and 3.5 M NEt_4_Cl (Np/NEt_4_, blue line); Raman spectra of a 3.5 M NEt_4_Cl solution (green line) and of the crystalline product [NMe_4_]Cl[NpO_2_Cl(H_2_O)_4_] (**3**) (orange line). The intensities of all spectra are scaled to facilitate comparison

Raman spectra confirm the hypothesis that additional Np^V^O_2_^+^ species form in the TAACl solutions, as illustrated by the spectra for Np^V^ solutions in which LiCl was gradually replaced with NEt_4_Cl (Fig. 2). For this experiment, NEt_4_Cl was substituted for NMe_4_Cl to avoid spectral interference from the [NMe_4_]^+^ cation (*ν*_1 _≈ 759 cm^−1^). In solutions of 3.5 M LiCl, the Raman spectrum of Np^V^ exhibits the characteristic Np^V^O_2_^+^ *ν*_1_ band at 767 cm^−1^, which originates from symmetric stretching of the O=Np^V^=O unit of the hydrated cation, [NpO_2_(H_2_O)_5_]^+^ (Fig. 2)^29^. With increasing NEt_4_Cl concentrations, the Raman spectra reveal the ingrowth of a vibrational band at ≈736 cm^−1^ (Fig. 2). This band occurs as a low-frequency shoulder on the Np^V^O_2_^+^ *ν*_1_ band, indicating that the presence of TAA^+^ cations induces the formation of Np^V^ species with weaker Np–O_yl_ bonding.

There are two known mechanisms that could cause a red shift of the actinyl *ν*_1_ vibrational band. First, weakening of the Np^V^–O_yl_ bond could be due to the inner-sphere coordination of electron-donating chloride ligands in the Np^V^ equatorial plane^24,30,31^. Alternatively, the formation of [O=Np^V^=O···M^*n*+^] complexes (M^*n*+^ = highly charged metal cation) could also weaken the Np–O_yl_ bond and decrease the *ν*_1_ frequency, as previously observed in dimeric Np^V^ CCI complexes^32^.

However, in contrast to the spectra of known aqueous [Np^V^O_2_Cl_*n*_(H_2_O)_*m*_]^(*n*–1)^ or [O=Np^V^=O···M^*n*+^] complexes^11,15,23^, the Vis–NIR absorption spectra of the Np^V^–TAACl solutions (Supplementary Figure 6) do not show the ingrowth of peaks at wavelengths higher than the characteristic Np^V^ 5*f* → 5*f* transition at 980.2 nm^33^, suggesting that the interactions between Np^V^O_2_^+^ cations and other constituents in the TAACl solutions, which include Cl^−^ and  TAA^+^ ions and H_2_O molecules, deviate from any interactions previously reported. Instead, the absorption spectra for 5 mM Np^V^ dissolved in 1, 3, and 5 M NMe_4_Cl solutions exhibit a small, but significant (up to 2 nm) gradual blue shift of the 980.2 nm band (Supplementary Discussion and Supplementary Figure 6). No noticeable shift of the 980.2 nm band was observed in the absorption spectra for the 5 M LiCl solutions. These results are consistent with findings from the electroanalytical experiments, confirming a negligible impact of LiCl on Np^V^ chemistry in solution.

### Syntheses and structures of representative Np compounds

How do Np^V^O_2_^+^ cations interact with other solution constituents, particularly TAA^+^ cations, and how do these interactions stabilize higher-oxidation-state neptunyl species? To answer these questions, we crystallized potential neptunyl solution species by evaporation of acidic chloride solutions containing either Li^+^ or TAA^+^ cations. Subsequently, the solid products were characterized using single-crystal X-ray diffraction and Raman spectroscopy. The evaporation experiments reported here were conducted under conditions comparable to previously reported reactions containing only Np^V^O_2_^+^ and HCl^16,34^, for which open-air evaporation yields a range of products, including the CCI compound, (Np^V^O_2_)Cl(H_2_O)_2_, a mixed-valent Np^IV/V^ compound, and other unidentified green phases^16,34^.

**Fig. 3:**
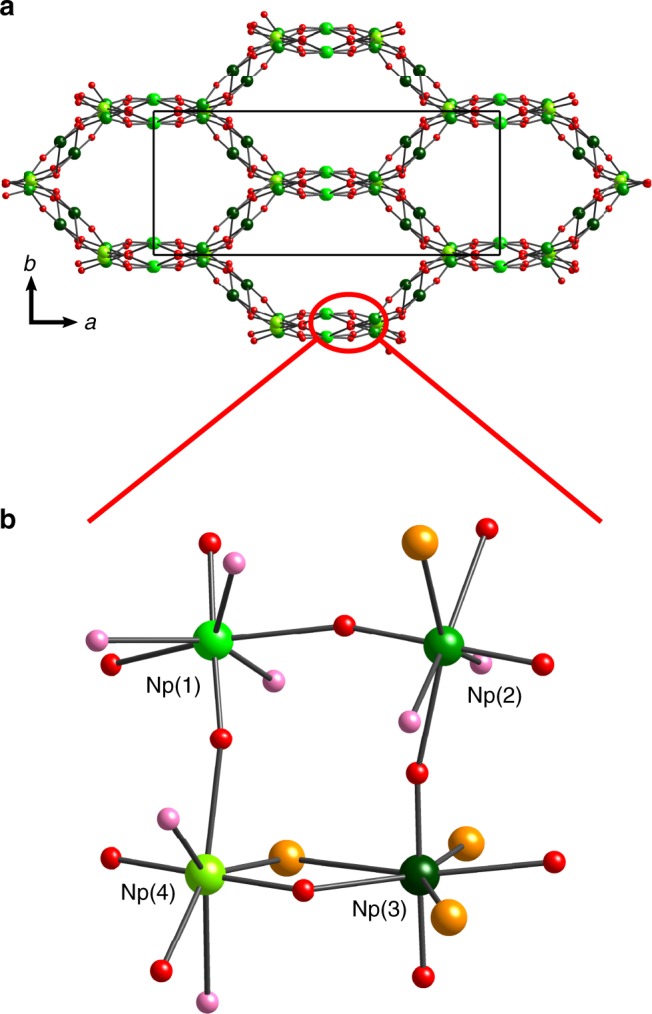
Structure of (NpO_2_)_4_Cl_4_(H_2_O)_7_ (**1**). **a** Projection of the three-dimensional network of cation−cation-bonded Np^V^O_2_^+^ square nets, with open channels along the [0 0 1] direction. Chloride anions and water molecules that reside inside the channels are omitted for clarity. **b** Local coordination environments and connectivities of four crystallographically unique Np^V^O_2_^+^ cations in the structure of **1**. Half of the disordered Cl and O_w_ positions are omitted for clarity. Green, red, pink, and orange spheres represent Np, O_yl_ (yl = actinyl), O_w_ (w = water), and Cl atoms, respectively

The open-air evaporation of Np^V^/HCl solutions containing Li^+^ yields similar products, confirming that Li^+^ does not influence the redox behavior of Np^V^ or the formation of CCI complexes in solution. All of the green products that form from the evaporation of Np^V^/Li/HCl solutions, including the compound, (NpO_2_)_4_Cl_4_(H_2_O)_7_ (**1**), have Raman signatures matching those of products from Li-free Np^V^/HCl reactions (Supplementary Discussion and Supplementary Figure 13). Although the disproportionation of Np^V^ to Np^IV^ and Np^VI^ is also possible during evaporative syntheses from acidic solutions, no evidence for the formation of Np^VI^ compounds was observed. The structure of **1** is closely related to that of the dihydrate, (Np^V^O_2_)Cl(H_2_O)_2_^16^, both of which adopt a 3-D CCI network of squarely arranged Np^V^O_2_^+^ cations with open channels filled by Cl^–^ anions and H_2_O molecules (Fig. 3a, Supplementary Discussion, Supplementary Figure 9, and Supplementary Figure 10). With a pentagonal bipyramidal geometry, each Np^V^O_2_^+^ cation in the dihydrate and in **1** is coordinated by five ligands in the equatorial plane, which include two yl–O atoms, zero to three H_2_O molecules, and zero to three Cl^–^ anions (Fig. 3b and Supplementary Figure 9). The Np–O_yl_ distances (Supplementary Table 3) and *ν*_1_ stretching frequencies for the dihydrate and **1** are also comparable at approximately 1.845 Å and 672 cm^–1^ (Supplementary Discussion).

In contrast, during solution evaporation in open air, the presence of [NMe_4_]^+^ in the Np^V^/HCl solutions drastically alters neptunium’s coordination and redox chemistry. Unlike the solid products that formed during the evaporation of solutions containing Li^+^, no Np^V^ CCI and no Np^IV^ compounds formed during the evaporation of solutions containing [NMe_4_]^+^. Instead, yellow–green crystals of [NMe_4_]_2_[Np^VI^O_2_Cl_4_] (**2**) form readily upon solution evaporation in air, indicating that the presence of [NMe_4_]^+^ cations inhibits the formation of CCI compounds and promotes Np^V^ oxidation to Np^VI^. This finding is consistent with our voltammetry data that clearly demonstrate enhanced stabilization of Np^VI^ relative to Np^V^ in the presence of [NMe_4_]^+^ cations.

**Fig. 4:**
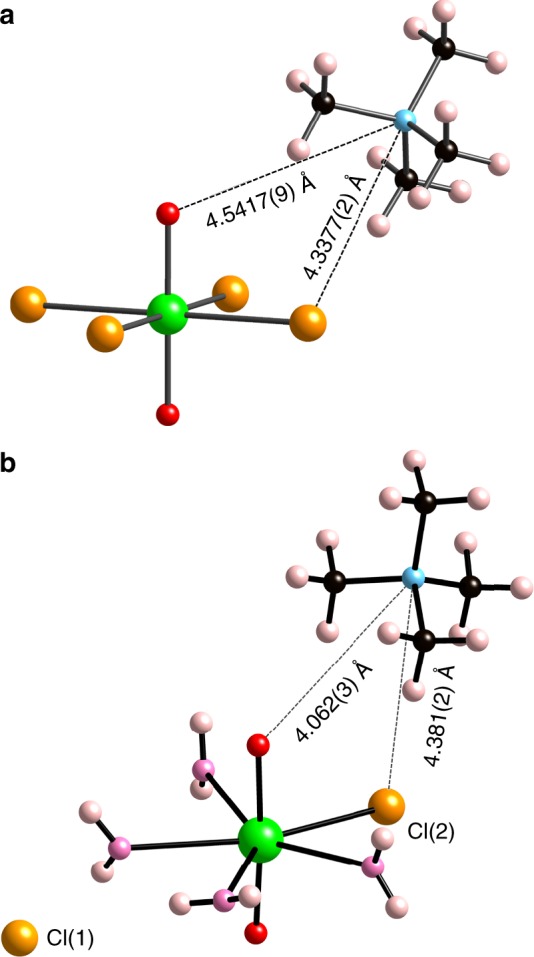
Structures of Np^VI^– and Np^V^–[NMe_4_] chlorides. **a** Local connectivities between one crystallographically unique Np^VI^O_2_^2+^ cation, one Cl^−^ anion, and one [NMe_4_]^+^ cation in the structure of [NMe_4_]_2_[NpO_2_Cl_4_] (**2**). **b** Local connectivities between one crystallographically unique Np^V^O_2_^+^ cation, two Cl^−^ anions, four water molecules, and one [NMe_4_]^+^ cation in the structure of [NMe_4_]Cl[NpO_2_Cl(H_2_O)_4_] (**3**). Green, red, pink, orange, blue, black, and beige spheres represent Np, O_yl_, O_w_, Cl, N, C, and H atoms, respectively. H-bonding is omitted for clarity

The structure of compound **2**, which consists of molecular [Np^VI^O_2_Cl_4_]^2−^ complexes and [NMe_4_]^+^ cations (Fig. 4a, Supplementary Discussion, and Supplementary Figure 11), also provides a possible model for the interplay between oxidized Np cations and other solution constituents in the electrochemical experiments (Fig. 4a). In fact, the Np^VI^O_2_^2+^ unit in the structure of **2** appears to be strongly perturbed by its surrounding environment, which includes Cl^−^ and [NMe_4_]^+^ ions. More specifically, Np−O_yl_ distances within the tetragonal bipyramidal [Np^VI^O_2_Cl_4_]^2−^ units in **2** are 1.765(3) Å, which are much longer than those for [Np^VI^O_2_(H_2_O)_5_]^2+^ complexes in the structure of [NpO_2_(H_2_O)_5_](ClO_4_)_2_ (1.7479(9) and 1.740(1) Å)^35^. The *ν*_1_ symmetric stretching band for O=Np^VI^=O moieties in **2** occurs at 797 cm^−1^ (Supplementary Figure 13), which is approximately 60 cm^−1^ less than that for free [Np^VI^O_2_(H_2_O)_5_]^2+^ complexes in solution (855 cm^−1^). This finding confirms weakening of the Np^VI^–O_yl_ bond in **2**, which has been attributed to the coordination of four chloride (electron-donating) ligands in the Np^VI^O_2_^2+^ equatorial plane in similar compounds. Additional non-covalent interactions between yl–O atoms and [NMe_4_]^+^ cations and between coordinated Cl^–^ anions and [NMe_4_]^+^ cations are apparent, which may also affect the electronic structure of the Np^VI^O_2_^2+^ cations in **2**. In total, each discrete [Np^VI^O_2_Cl_4_]^2−^ anion attracts eight neighboring [NMe_4_]^+^ cations through electrostatic interactions with O_yl_ and Cl^−^ moieties with O_yl_−N and Cl−N distances of 4.5417(9) Å and 4.3377(2)–4.5617(3) Å (Fig. 4a, Supplementary  Discussion, and Supplementary Table 4). Additionally, each O_yl_ and Cl atom potentially participates in four H-bonds with the methyl group of four neighboring [NMe_4_]^+^ cations, with donor–acceptor distances ranging from 3.385(3) Å (C−H⋅⋅⋅O_yl_) to 3.686(2)–3.901(3) Å (C−H⋅⋅⋅Cl).

To isolate potential Np^V^ species present in TAACl solutions, we repeated the above evaporation experiment in the absence of air to exclude the potential oxidizer, O_2_. As a result, only teal crystals of [NMe_4_]Cl[Np^V^O_2_Cl(H_2_O)_4_] (**3**) were obtained. In addition to discrete [NMe_4_]^+^ and Cl^–^ ions, compound **3** contains molecular actinyl monochloride complexes, [Np^V^O_2_Cl(H_2_O)_4_], which confirms the existence of Np^V^–monochloro species in TAACl solutions (Fig. 4b, Supplementary Discussion, and Supplementary Figure 12). No other Np chemistry (i.e., formation of CCI or Np^IV^ compounds) was observed during this evaporation experiment, confirming that [NMe_4_]^+^ cations inhibit the formation of Np CCI complexes and suggesting that O_2_ is the oxidant responsible for the Np^V^ oxidation observed during the evaporation of NMe_4_Cl solutions in open air.

In addition, the structure of **3** provides crucial evidence supporting the substantial impact of neptunium-extended coordination environments on Np^V^–O_yl_ bonding. This is evidenced by the elongated Np^V^–O_yl_ distances (1.838(2) and 1.843(2) Å) and the red-shifted symmetric stretching frequency (*ν*_1_ = 741 cm^−1^) in **3** (Fig. 2), compared with the distances and symmetric stretching frequencies observed in other non-CCI compounds, such as Na_3_(Np^V^O_2_)(SeO_4_)_2_(H_2_O) (Np–O_yl_ = 1.813(3) Å and 1.831(3) Å; *ν*_1_ = 773 cm^−1^)^36^. Considering the weaker donating capacity of Cl^−^ anions compared with oxoanions^24,30^, such as selenate, the weakened Np–O_yl_ bonding in **3** results from the non-covalent interactions between Np^V^O_2_^+^ cations and [NMe_4_]^+^ cations and H_2_O ligands. Each [NpO_2_Cl(H_2_O)_4_] complex in the structure of **3** bonds to five [NMe_4_]^+^ cations through O_yl_/Cl(2)^–^⋅⋅⋅[NMe_4_]^+^ electrostatic interactions and through potential H-bonds between Cl(2) atoms and methyl groups (Supplementary Discussion and Supplementary Table 5). More specifically, each yl–O atom attracts one [NMe_4_]^+^ cation with O_yl_−N distances of 4.062(3) or 4.660(3) Å and each Cl(2)^−^ anion attracts four [NMe_4_]^+^ cations with Cl−N distances in the range of 4.184(2)−5.156(2) Å. In addition, each yl–O atom participates in two H-bonds with the H_2_O ligands from surrounding [NpO_2_Cl(H_2_O)_4_] complexes, with O_w_−H⋅⋅⋅O_yl_ distances of 2.733(3)−2.859(3) Å. Interestingly, the neptunyl *ν*_1_ band for the pentagonal bipyramidal [Np^V^O_2_Cl(H_2_O)_4_] complexes in **3** matches the frequency of the shoulder peak observed in the Raman spectrum of the 0.12 M Np^V^/3.5 M NEt_4_Cl solution (Fig. 2). This finding indicates that the Np^V^/NEt_4_Cl solution contains Np^V^O_2_^+^ species that have similar structural properties and that participate in similar non-covalent interactions as the Np^V^O_2_^+^ species in **3**.

### Molecular dynamics simulations

The non-covalent extended-sphere interactions between Np^V^ and TAA^+^ cations and the influence of these interactions on the first coordination sphere of Np^V^ are further supported by all-atom molecular dynamics (MD) simulations. All MD simulations were performed on 0.1 M Np^V^ or Np^VI^ solutions at pH = 2 in the presence of 5 M LiCl or 5 M NMe_4_Cl (Supplementary Tables 6 and 7, and Supplementary Figures 14, 15, and 16). Consistent with our experimental results, the MD simulations confirm that neptunyl species in 5 M LiCl solutions are dominated by hydrated neptunyl complexes, with only weak Np–Cl coordination (Table 1). Surrounding these neptunyl hydrates, there are only 0.3 and 0.5 Li^+^ cations, on average, indicating roughly negligible interactions between the neptunyl and Li^+^ cations. In contrast, both Np^V^ and Np^VI^ exhibit enhanced chloride ligation in the presence of 5 M NMe_4_Cl. Specifically, substantial quantities of neptunyl^V^ mono- and dichlorides and neptunyl^VI^ di- and trichlorides formed under the tested conditions (Table 1). These neptunyl–chloride complexes are embedded inside a cage of six (average) [NMe_4_]^+^ cations, each of which is located at approximately 6 Å from the Np centers, indicating significant non-covalent interactions between the [NMe_4_]^+^ electrolyte cations and the Np first-coordination sphere. These arrangements are comparable to those observed in the structures of **3** and **2** (Fig. 4), where each neptunyl–chloride complex bonds five or eight [NMe_4_]^+^ cations via electrostatic and potential H-bonding interactions with the yl–O atoms or Cl^−^ anions, with Np–N distances of 5.292(4)–7.424(2) Å and 5.3745(3) Å for Np^V^ and Np^VI^, respectively.

**Table 1:** Average coordination environment surrounding Np^V^O_2_^+^ and Np^VI^O_2_^2+^ cations in LiCl or NMe_4_Cl solutions determined from all–atom MD simulations^a^

System	O_w_ *r* (Å)	O_w_ CN^b^	Cl^−^ *r* (Å)	Cl^−^ CN^b^	Li^+^/[NMe_4_]^+^ *r* (Å)	Li^+^/[NMe_4_]^+^ CN^b^	*ε*_r_^c^
Np^V^/LiCl	2.548	4.4	2.842	0.4	4.44	0.3	38.8
Np^V^/NMe_4_Cl	2.530	2.4	2.818	1.8	5.55	5.9	21.6
Np^VI^/LiCl	2.476	3.8	2.758	1.1	4.58	0.5	39.0
Np^VI^/NMe_4_Cl	2.467	2.0	2.718	2.4	6.14	5.9	21.3

^a^Simulation conditions: 0.1 M Np, pH 2, 5 M LiCl or NMe_4_Cl

^b^CN = coordination number

^c^Relative permittivity

## Discussion

On the basis of the experimental and simulation results, we propose that TAA^+^ cations promote the formation of inner-sphere Np^V^–chloride complexes in solution. The formation of such complexes is expected to decrease the measured Np^VI^/Np^V^ electrode potential because Np^VI^O_2_^2+^ cations form stronger complexes with electron-donating chloride ligands than Np^V^O_2_^+^ cations^19,23^. Our combined data, particularly the crystallization of [NMe_4_]Cl[Np^V^O_2_Cl(H_2_O)_4_] (**3**) and the enhanced neptunyl–chloride complexation observed in the MD simulations for NMe_4_Cl solutions, suggest that monochloro–Np^V^ complexes coexist with [NpO_2_(H_2_O)_5_]^+^ complexes in TAACl solutions. However, existing thermodynamic data reveal that the free energy difference expected as a result of the formation of monochloro–neptunyl complexes (ΔΔ*G* = −3.94 kJ mol^−1^, Supplementary Table 8) is inadequate to account for the large thermodynamic stabilization of Np^VI^ observed in 5 M NMe_4_Cl (Δ*E*_1/2_ ≈ −220 mV → ΔΔ*G* = –22.2 kJ mol^−1^, Supplementary Table 8). The formation of higher-order neptunyl–chloride complexes in solution is also possible, as indicated by the structure of **2** and the MD results, and may further decrease the Np^VI^/Np^V^ electrode potential^37^. The corresponding thermodynamic data for higher-order neptunyl–chloride complexes are unknown, but a decrease of the Np^VI^/Np^V^ electrode potential less than 220 mV would be expected, considering the weak electron-donating capability of the chloride ligand^24,30^.

In addition to the Np^VI^ stabilization conferred upon chloride complexation, the extended-sphere association with TAA^+^ cations, forming [Np^V^O_2_Cl_*n*_(H_2_O)_*m*_]^(1–*n*)^•••TAA_*x*_^+^ groups, further decreases the Np^VI^/Np^V^ electrode potential. For homogeneous 2 and 5 M NMe_4_Cl solutions, the calculated separation between the centers of [NMe_4_]^+^ and Cl^−^ ions is about 7.5 and 5.5 Å, which are close to the N−Cl distances for solvent-separated (7.5Å) and contact (5.0 Å) [NMe_4_]^+^−Cl^−^ ion pairs in aqueous solution^38^. As a result, interactions between TAA^+^ cations and free Cl^−^ anions and between TAA^+^ cations and the Cl^−^ anions of [Np^V^O_2_Cl_*n*_(H_2_O)_*m*_]^(1−*n*)^ complexes are expected under the solution conditions employed herein. Considering the structure of **3** (Fig. 4) and the MD results (Supplementary Figure 16), we also expect electrostatic and potential H-bonding interactions between TAA^+^ cations and yl–O atoms in solution. As a whole, it appears that yl–O atoms and coordinated Cl^−^ ligands are both key players mediating the association of neptunyl and TAA^+^ cations. To separate the synergetic effect of yl–O atoms and Cl^−^ ligands, similar experiments with the non-complexing ClO_4_^–^ ligand were planned but could not be executed because of the much lower aqueous solubility of TAAClO_4_ compared with TAACl salts.

As a final note, we must also consider the chemical and structural properties of water itself, and the important role that water plays in influencing the electrochemical stabilities of neptunyl complexes. As indicated in the structure of **3** and other reported evidence in the literature^37,39^, water can act as a H-bond donor to the yl–O atoms, particularly those of Np^V^O_2_^+^, in a manner competing with TAA^+^ cations. With increasing concentrations of TAACl, the concentration of water decreases. This effectively decreases the H-bonding interactions between water molecules and neptunyl cations and decreases the relative permittivity of the solutions (Table 1), facilitating stronger interactions between neptunyl cations and Cl^−^ and TAA^+^ ions.

The divergence in the reactivity of Li^+^ and TAA^+^ cations toward the neptunyl first-coordination sphere in aqueous solutions demonstrated in this study can be attributed to the different solvation properties of the electrolyte cations. In aqueous solutions, Li^+^ cations are strongly hydrated (Δ*H*_hydration_ = −530 kJ mol^−1^)^40^, such that their charge is effectively shielded from the neptunyl–O atoms and Cl^−^ ions. However, TAA^+^ cations, which are only weakly hydrated in solution (Δ*H*_hydration _≈ −200 kJ mol^−1^)^40^, are expected to more actively engage with the neptunyl–O atoms and Cl^−^ ions via electrostatic interactions. Additionally, potential H-bonding via the alkyl groups may further enhance the association of TAA^+^ cations and neptunyl complexes in solution. Therefore, despite their lower charge density, compared with Li^+^ cations, TAA^+^ cations are expected to associate with Np^V^O_2_^+^ cations in aqueous solutions. These phenomena are well illustrated by the structures of **2** and **3** and by the MD simulation results. The observed differences in neptunyl chemistry in LiCl vs. TAACl solutions are consistent with our recent findings that demonstrate a correlation between countercation (A^+ ^= Li^+^, Na^+^, K^+^, Rb^+^, Cs^+^, NH_4_^+^, or NR_4_^+^ (R = Me, Et, and Bu)) hydration enthalpies and the formation of redox-inactive Th–nitrato molecular complexes from aqueous solution, wherein hydrophobic countercations (e.g., TAA^+^ cations) tend to associate with more anionic Th−nitrato molecular complexes via stronger non-covalent interactions in solid structures^41^.

These results highlight the importance of ion hydration properties in regulating the interactions between oxocations and electrolyte cations in solution. The competition between ion solvation and ion association has been well documented for oppositely charged ions such as A^+^ (A = alkali metal and tetra-*n*-alkylammonium) and Cl^−^^8^. Weak ion–solvent interactions also appear to be essential for ion association between neptunyl species and TAA^+^ cations in aqueous solutions. Related phenomena have been reported for other oxocation and metal ion systems, supporting the fundamental importance of these underlying chemical principles. For example, stronger interactions between Np^V^O_2_^+^ and highly charged metal cations, such as Al^3+^ and Fe^3+^, are observed in mixed aqueous polar–organic solvents than in aqueous media because ion–solvation interactions are weaker in the mixed solvents^42^. Other metal–oxo complexes with similar extended coordination environments, such as those of iron–^43^, manganese–^5,6^, and uranium–oxocations^3,4^, have been isolated in organic solvents, where weak ion–solvent interactions are negligible compared to the strong bonding between oxo groups and extended-sphere metal cations.

The influence of hydrophobic, low-charge density TAA^+^ cations on the electrochemical properties of neptunyl species in aqueous chloride media arises from a mechanism different than that reported previously for studies involving strong Lewis acids, which have a comparatively higher charge density. These previous studies suggest that direct bonding between Lewis acids, such as Ca^2+^ or Y^3+^, and oxo groups tends to increase the electrode potentials of oxocations as a function of increasing Lewis acidity^6^. In contrast, TAA^+^ cations associate with neptunyl cations via non-covalent interactions with both the neptunyl oxo groups and equatorial ligands. This association influences ligation within the metal cation’s first coordination sphere, driving enhanced coordination of anionic Cl^−^ ligands to the Np^V/VI^ centers. The sum of all these interactions, namely the inner-sphere coordination with Cl^–^ anions and the outer-sphere association with TAA^+^ cations, causes a significant decrease of the Np^VI^/Np^V^ electrode potential to favor Np^VI^.

Overall, this work opens an avenue of using non-innocent electrolyte cations to control the redox properties and the first-coordination-sphere ligation of metal cations. Furthermore, we highlight that the observed influence of TAA^+^ cations on the coordination and redox chemistry of neptunyl cations is important in actinide separation processes in which quaternary ammonium cations (e.g., TAA^+^) are widely employed as anion exchange extractants or in room-temperature ionic liquids (RTILs)^44–46^. In fact, higher-order (anionic) actinide complexes, including chloride complexes, which would not usually be considered dominant aqueous species based on solution ligand concentrations, are preferentially associated with quaternary ammonium cations in these processes^45^. This study provides significant insight into the underlying principles regarding the interactions between actinide species and quaternary ammonium cations, which will aid in optimizing actinide separation processes and tuning the properties of RTILs.

## Methods

### Caution!

^237^Np is an α- and γ-emitting radioisotope and is considered a health risk. Its use requires appropriate infrastructure and personnel trained in the handling of radioactive materials.

### Stock solution preparation

Solutions of Np^V^ in 1M HClO_4_ were purified using a cation-exchange column containing Dowex-50-X8 resin. After purification, Np was precipitated using NaOH, washed with water, and redissolved in 1M HCl. The procedure was used to prepare two separate stock solutions, one for solid-product syntheses and Raman spectroscopy and one for electrochemistry experiments. Identification of the characteristic near-infrared absorption band for Np^V^ at ≈980 nm, and the absence of absorption bands characteristic for other Np oxidation states, confirmed that both Np/HCl stock solutions contained only Np^V^ (Supplementary Figure 1)^47^. The final purified stock solutions contained 0.24 M or 0.1 M Np^V^, as determined using liquid scintillation counting (LSC). LiCl (Fisher, >99.0%), NMe_4_Cl (Acros Organics, >98%), and NEt_4_Cl (Sigma, >98%) were used as obtained. ACl (A = Li^+^, [NMe_4_]^+^, and [NEt_4_]^+^) stock solutions of varying concentrations were prepared by dissolving ACl in deionized H_2_O.

### Voltammetry

Cyclic and differential pulse voltammetry (CV, DPV) data for 5 mM Np^V^ solutions in various static electrolytes were collected at room temperature in the absence of oxygen using a BAS 100B electrochemical workstation, a graphite working electrode, a graphite auxiliary electrode, and a Ag/AgCl (3 M NaCl) reference electrode, which has a redox potential of +0.200 V vs. SHE at 25 °C. All Np solutions used for the electrochemical experiments were prepared individually by mixing an aliquot of the 0.1M Np^V^ stock solution with appropriate volumes of ACl stock solutions and H_2_O to yield the solutions listed in Supplementary Table 1. The Np^V^ concentrations of select sample solutions were verified using LSC.

### Visible–near-infrared (Vis–NIR) absorption spectroscopy

Vis–NIR spectra (400–1300 nm) were collected for diluted Np^V^ stock solutions in standard 1 cm path-length polystyrene cuvettes (Fisher) and for select 5 mM Np^V^ electrochemistry solutions in 0.2 cm path-length micro-volume polystyrene cuvettes (Eppendorf, UVette) using an Olis-modernized Cary 14 spectrophotometer.

### Synthesis of (NpO_2_)_4_Cl_4_(H_2_O)_7_ (1)

A 25 μL aliquot of the 0.24 M Np^V^ stock solution (in ≈1 M HCl) and 6 μL of a 1 M LiCl solution were combined in a 2 mL glass vial and covered by parafilm with small pin holes. The solutions were left in a vented hood and allowed to slowly evaporate over three months to complete dryness. Solid products included green crystals of **1**, a green amorphous solid, and colorless salts (Supplementary Figure 7a).

### Syntheses of [NMe_4_]_2_[NpO_2_Cl_4_] (2) and [NMe_4_]Cl[NpO_2_Cl(H_2_O)_4_] (3)

A 10 μL aliquot of the 0.24 M Np^V^ stock solution and 5 μL of a 1 M NMe_4_Cl solution were combined in a 2 mL glass vial and covered by parafilm with small pin holes. The solutions were left in a vented hood and allowed to slowly evaporate to complete dryness. Solid products included up to millimeter-sized yellow–green crystals of **2** and colorless salts (Supplementary Figure 7b). Slowly evaporating the same solution in an apparatus excluding air resulted in teal crystals of **3** and colorless salts (Supplementary Figure 7c).

### Structure determinations

Single-crystal X-ray diffraction data for (NpO_2_)_4_Cl_4_(H_2_O)_7_ (**1**), [NMe_4_]_2_[NpO_2_Cl_4_] (**2**), and [NMe_4_]Cl[NpO_2_Cl(H_2_O)_4_] (**3**) were collected with the use of graphite-monochromatized MoK*α* radiation (*λ* = 0.71073 Å) at 100 K on a Bruker APEXII diffractometer. The crystal-to-detector distance was 5.00 cm. Data were collected by a scan of 0.3° in *ω* in groups of 600 frames at φ settings of 0°, 90°, 180°, and 270°. The exposure time was 25 s/frame, 15 s/frame, and 20 s/frame for **1**, **2**, and **3**, respectively. The collection of intensity data as well as cell refinement and data reduction were carried out with the use of the program APEX2^48^. Absorption corrections as well as incident beam and decay corrections were performed with the use of the program SADABS^48^. The structures were solved with the direct-methods program SHELXS and refined with the least-squares program SHELXL^49^. Further information is provided in the Supplementary Methods, with select crystallographic data listed in Supplementary Table 2.

### Raman spectroscopy

Raman spectra of Np containing solid samples and solutions were collected on a Renishaw inVia Raman Microscope with a circularly polarized excitation line of 532 nm. Due to the radiological hazards associated with ^237^Np, each solid sample or 2 μL solution sample was placed on a glass drop slide covered with a transparent coverslip, which was sealed to the slide using epoxy. Numerous spectra from multiple spots were collected on multiple samples for each compound and on each solution to ensure sample homogeneity. All Np solutions used for Raman spectral analysis were prepared individually by mixing an aliquot of the 0.24 M Np^V^ stock solution with appropriate volumes of ACl stock solutions and H_2_O.

### Molecular dynamics simulations

All-atom explicit-solvent MD simulations were performed using the package GROMACS (5.0.7)^50^. Force field parameters for Np^VI^O_2_^2+^ and Np^V^O_2_^+^ ions and the recommended SPC/E water model given by Pomogaev et al.^51^ were used in all simulations. Additional details and calculated radial distribution functions are given in the Supplementary Methods and in Supplementary Figures 14 and 15.

## Supplementary information

Supplementary Information

## Data Availability

All data supporting this work are available within this manuscript or its associated Supplementary Information, or from the corresponding author upon reasonable request. Additionally, crystallographic data for the compounds reported herein have been deposited at the Cambridge Crystallographic Data Centre (CCDC), under deposition numbers CSD 1878175-1878177. These data can be obtained free of charge from the CCDC via www.ccdc.cam.ac.uk/data_request/cif.
