# Supplementary material for: Ion association with tetra-n-alkylammonium cations stabilizes higher-oxidation-state neptunium dioxocations
Source: Nat Commun. 2019 Jan 4;10:59. doi: 10.1038/s41467-018-07982-5 (PMC6320366; doi:10.1038/s41467-018-07982-5)
Supplement: Supplementary file 1 — Supplementary Information [file 41467_2018_7982_MOESM1_ESM.pdf]

Supplementary Information

Ion Association with Tetra-*n*-alkylammonium Cations Stabilizes Higher–Oxidation–State Neptunium Dioxocations

Estes *et al*.

Supplementary Methods.

Crystal Structure Solution and Refinement. For all structures, assignments of most non-hydrogen atoms were straightforward, based on their electron densities and coordination environments.

In the structure refinement of **1**, substantial residual electron densities (~ 4 e·Å^{-3}) were located in the difference Fourier maps mostly at < 1.0 Å from the closest Np positions. Attempts to include minor twin components or increase the absorption coefficient did not improve the refinement results. Cl(4) and Cl(5) atoms were found to be disordered with O_w(7) and O_w(8) (w = H_2O), respectively, which are located at ~ 0.6 Å from the closest Cl positions. The constrained refinement with a unity occupancy for each set of two positions led to occupancies of 0.60(2)/0.40(2) for Cl(4)/O_w(7) and 0.42(2)/0.58(2) for Cl(5)/O_w(8). The refinement statistics did not change when the occupancies of Cl(4)/O_w(7) and Cl(5)/O_w(8) were fixed at 0.6/0.4 and 0.4/0.6 in the final refinement, so that charge balance of NpO_2^+ and Cl^- ions is achieved. No supercell reflections were observed as demonstrated in the synthesized precession images (Supplementary Figure 8). Therefore, the distribution of Cl(4)/O_w(7) and Cl(5)/O_w(8) atoms within the structure of **1** is random. H atoms could not be identified from the difference Fourier maps, so they were not included in the structural refinements of **1**. In the structure refinement of **2**, the H atoms of methyl groups were identified in the difference Fourier maps and their positional and isotropic displacement parameters were refined without any constraints. The largest residual density (1.866 e·Å^{-3}) in the difference Fourier maps is located at ~3.32 Å and ~3.28 Å from the closest chloride ligand and methyl carbon. Further examination of all residual electron density peaks suggests that the structure may include minor disorder, in which the O(1) and the Cl(1) atoms are exchanged. The largest density peak may then represent an alternative position for the $[NMe_4]^+$ cation. However, this disorder could not be modeled with confidence, as anisotropic thermal parameters for the exchanged O(1) and Cl(1) atoms became non-positive definite and the alternative position of the $[NMe_4]^+$ cation was unstable. Alternatively, the largest residual density peak may represent a solvent water bound to Cl^- and $[NMe_4]^+$ ions via hydrogen bonds; however its partial occupancy is too low to refine confidently. Similar behavior has been observed in the structure refinements of isostructural U and Pu compounds.[1] In the structure refinement of **3**, two non-merohedral twin components were included in the final refinement, with the second twin component rotated from the first by 179.6°. The two twin domains are related by the twin law [–1.000 0.000 –0.003 –0.563 1.000 –0.482 0.012 0.002 –1.001]. All H atoms bonded to O_w and methyl C were located in the difference Fourier maps. H positions for the water molecules were refined using direct O−H and H−H distance restraints of 0.83 Å and 1.40 Å, respectively. H atoms coordinated to C were placed in idealized positions. Isotropic displacement parameters of hydrogen atoms were constrained at 1.20 times or 1.5 times the U_{eq} value of the O_w and C to which the hydrogen bonds.

The final refinement included anisotropic displacement parameters for all non-hydrogen atoms. The program STRUCTURE TIDY[2] was used to standardize the positional parameters. Additional experimental details are given in Supplementary Table 2 and in the CIFs.

All–Atom Explicit–Solvent Molecular Dynamics Simulations. All-atom MD simulations were performed using the package GROMACS (5.0.7)[3] using force field parameters for NpO_2^{2+} and NpO_2^+ as reported by Pomogaev et al.[4] using the SPC/E water model. The structures of the SPC/E water model were constrained using the SETTLE algorithm.[5] The force field parameters of H_3O^+ were recently reported by van Keulen et al.[6] The force field parameters of all the other molecules are from the original CHARMM force field.[7, 8]

In the present work, two neptunyl ions were investigated, NpO_2^{2+} and NpO_2^{+}, each in 5M LiCl and 5M NMe_4Cl. All the simulations were performed with an H_3O^+ concentration of 0.01 M, corresponding to the experimental condition of pH = 2. See Supplementary Table 6 for the compositions. The initial structures were built using the package Packmol.[9]

The energy of the initial structure was first minimized using the steepest descent algorithm. An equilibration of 10 ps using the NTV ensemble (constant number of particles, temperature and volume) was followed by further equilibration of 0.1 ns using the NTP ensemble (constant number of particles, temperature and pressure). The production simulations were subsequently performed. Neighbor searching was done up to a cutoff distance of 1.2 nm. The short-range Coulomb interactions were calculated up to 1.2 nm with the smooth particle mesh Ewald (PME) method for the long-range electrostatic interactions with a grid real spacing of 0.12 nm and six order interpolation.[10, 11] The Lennard-Jones 12-6 potential was switched off from 1.0 nm to 1.2 nm with the force switching method. No long-range dispersion corrections were applied for the energy and pressure. These parameters have been recommended for the accurate reproduction of the original CHARMM simulations on lipid systems.[12] The simulation integration time step of 2 fs was employed with all the hydrogen-involved covalent bond lengths constrained using the LINCS algorithm.[13] Each of the production simulations was performed for a duration of 50 ns using a saving frequency of 10 ps for data collection. The first 2 ns simulation trajectory was discarded for the final analysis, as suggested by the calculations in the convergence of the system densities and potential energies.

Supplementary Discussion.

Cyclic Voltammetry. Cyclic voltammograms collected for 5 mM Np^V dissolved in 1 M and 5 M LiCl reveal increasing anodic and cathodic peak separations (ΔE_p) with increasing scan rate (v) (Supplementary Figure 2). Peak current ratios ($i_{p,c}/i_{p,a}$) in both the 1 M and 5 M LiCl solutions are less than one at all v and decrease gradually with increasing v. Together, these results indicate that the Np^{VI}/Np^V redox reaction in 1 M and 5 M LiCl is quasi-reversible and controlled by both charge-transfer and mass-transport. The DPV data (Supplementary Figure 2), which exhibit nearly symmetrical Gaussian peaks, confirm this finding. Furthermore, the Np^{VI}/Np^V electrode potentials, determined as the average half-wave potential ($E_{1/2}$) over all scan rates, in 1 M and 5 M LiCl (Supplementary Table 1) are equal (within error), although the charge-transfer kinetics between the two systems differ (Supplementary Figure 3).

Cyclic voltammograms collected for 5 mM Np^V dissolved in 1 M and 5 M NMe_4Cl (Supplementary Figure 4) differ substantially from each other and from the voltammograms collected for Np^V/LiCl solutions. In 1 M NMe_4Cl, increasing ΔE_p with increasing v and $i_{p,c}/i_{p,a}$ values less than one at all v indicate that the Np^{VI}/Np^V redox behavior is quasi-reversible, similar to the Np^{VI}/Np^V redox behavior in the LiCl solutions. However, the Np^{VI}/Np^V electrode potential measured in 1 M NMe_4Cl is significantly different than the electrode potentials measured in 1 M and 5 M LiCl (Supplementary Table 1). In fact, in 1 M NMe_4Cl, the Np^{VI}/Np^V electrode potential is shifted cathodically by approximately 30 mV, indicating a moderate stabilization of Np^{VI} compared to Np^V. When the NMe_4Cl concentration is increased to 5 M, the Np^{VI}/Np^V redox behavior changes even further (Supplementary Figure 4). For the 5 M NMe_4Cl system, the Np^{VI}/Np^V redox behavior is complicated both kinetically, as indicated by the increasing ΔE_p with increasing v, and chemically, as indicated by the increasing $i_{p,c}/i_{p,a}$ with increasing v. Most importantly, the Np^{VI}/Np^V electrode potential measured in 5 M NMe_4Cl is cathodically shifted by more than 200 mV compared with the electrode potentials measured in the Np^V/LiCl systems (Supplementary Table 1). The DPV data further confirm the quasi-reversibility and potential shifts for the Np^{VI}/Np^V couple in the 1 M and 5 M NMe_4Cl systems (Supplementary Figure 4). As $[NMe_4]^+$ concentrations increase, from 1 M to 3 M to 5 M, the Np^{VI}/Np^V electrode potential shifts proportionately to more cathodic potentials. This effect is observed regardless of whether ionic strength is maintained (using LiCl) across the concentration range (Supplementary Table 1). Similar behavior, namely a cathodic shift in the Np^{VI}/Np^V electrode potential, is also observed in CV data for Np^V dissolved in 3 M NEt_4Cl (NEt_4Cl concentration is limited by solubility) (Supplementary Figure 5).

Vis–NIR Spectroscopy. The electronic spectrum of the uncomplexed, hydrated $Np^VO_2^+$ cation exhibits an intense $5f \rightarrow 5f$ transition at 980.2 nm.[14] Changes in the symmetry of the $Np^VO_2^+$ unit, either through coordination to the yl–O atom or through coordination in the equatorial plane, can significantly alter both the intensity (molar absorptivity) and energy (wavelength) of this transition. For example, the formation of CCI complexes with the uranyl, UO_2^{2+}, cation[15, 16] or the formation of $[O{=}Np^V{=}O\cdots M^{n+}]$ complexes (M^{n+} = highly–charged metal cation)[17] causes a decrease in the intensity and energy (red-shift) of the Np^V 980.2 nm absorption band. Replacement of water in the equatorial plane of the $Np^VO_2^+$ cation with various ligands also typically causes a red-shift of the 980.2 nm band. However, if complexation in the equatorial plane creates inversion symmetry about the Np center, the $5f \rightarrow 5f$ transition becomes Laporte forbidden[14] and the complex will be optically silent in the Vis-NIR spectral range. Furthermore, calculated absorption spectra for several $[Np^VO_2(Cl)_x]^{1-x}$ (x = 0, 1, 3, 5) complexes support that the intensity of the Np^V $5f \rightarrow 5f$ transition is greatest for complexes containing five equatorial ligands.[14] With these considerations, the absorption spectra for Np^V dissolved in 1 M , 3 M, and 5 M NMe_4Cl (Supplementary Figure 6) compared

with that of the 0.1 M Np^V stock solution reveal that the coordination environment around the $Np^VO_2^+$ cation changes with increasing concentrations of NMe_4Cl, as evidenced by the small, but significant, blue-shift of the 980.2 nm band. Although the presence of optically silent $Np^VO_2^+$ complexes cannot be excluded, continued observation of intense $5f \rightarrow 5f$ transitions for Np^V/NMe_4Cl solutions further suggests that any new complexes formed do not possess inversion symmetry and likely retain five coordinating ligands in the $Np^VO_2^+$ equatorial plane. Despite the significant decrease in the measured Np^{VI}/Np^V electrode potential in 5 M NMe_4Cl, the corresponding absorption spectrum provides no evidence for the presence of Np in other oxidation states, not even as Np^{VI}. This indicates that although Np^{VI} is more stable in 5 M NMe_4Cl solutions than in, for example, 1 M $HClO_4$, the induced free-energy change ($\Delta\Delta G$ = –22.2 kJ mol^{-1} (see Supplementary Table 8) is not sufficient under these dilute concentrations to promote Np^V oxidation to Np^{VI}. However, increasing Np^V concentrations during evaporative syntheses in the presence of O_2 provide a sufficient thermodynamic driving force to promote oxidation to Np^{VI}.

Crystal Structures Descriptions. $(NpO_2)_4Cl_4(H_2O)_7$ (**1**) crystallizes in the monoclinic space group *C*2/*c*. As shown in Supplementary Figure 9, the structure of **1** contains four crystallographically unique nearly linear NpO_2^+ cations, each of which is coordinated by five ligands in the equatorial plane in a pentagonal bipyramidal geometry. More specifically, each NpO_2^+ cation is coordinated by two O_{yl} (yl = actinyl), zero to three O_w, and zero to three Cl atoms. The Np−O_{yl} distances range from 1.837(5) Å to 1.853(5) Å and O_{yl}−Np−O_{yl} angles range from 176.4(2) and 179.1(2)° (Supplementary Table 3). The equatorial Np−O_{yl}, Np−O_w, and Np−Cl distances are in the range of 2.385(5)−2.465(5) Å, 2.35(2)−2.607(9) Å, and 2.765(2)−2.892(2) Å, respectively. These interatomic distances within neptunyl polyhedra in **1** are comparable with those found in the structures of other Np^V chloride compounds. For example, the Np−O_{yl}, the equatorial Np−O_{yl}, Np−O_w, and Np−Cl distances for neptunyl(V) pentagonal bipyramids in the structure of $(NpO_2)Cl(H_2O)_2$ are found in the range of 1.840(2)−1.848(2) Å, 2.349(2)−2.471(2) Å, 2.439(2)−2.525(2) Å, and 2.7887(6)−2.8907(6) Å, respectively.[18]

The structure of **1** adopts a common neptunyl(V) CCI motif that is in a square arrangement, where each NpO_2^+ cation connects to four other units through CCIs (Supplementary Figure 10). More specifically, each NpO_2^+ cation acts as a center coordinated by two other units and as a ligand binds to two neighboring Np(V) centers. These "cationic square nets" of NpO_2^+ cations interpenetrate each other to create open channels along the [001], [1−10], and [110] directions, which are filled by chloride anions and water molecules (Supplementary Figure 10). Most NpO_2^+ cations in the structure of **1** are only connected to each other through CCIs and the Np−Np distances between those neighboring neptunyl units range from 4.1586(4) to 4.2454(3) Å. These distances are close to the corresponding values between adjacent Np^V centers (4.1845(3) to 4.2612(2) Å) found within "cationic square nets" of NpO_2^+ cations in the structure of $(NpO_2)Cl(H_2O)_2$.[18] In comparison, each $Np(3)O_2^+$ cation in the structure of **1** is further connected to one $Np(4)O_2^+$ cation through a bridging $Cl(3)^-$ anion in addition to the CCI. The Np−Np distances within these edge-sharing dimers of neptunyl(V) pentagonal bipyramids are 3.8589(4) Å, which are close to the value (3.885 (1) Å) found between two Np centers bridged by one O_{yl} and one Cl atoms in the structure of $(NpO_2)Cl(H_2O)$.[19]

The chemical formula of **1** can be rewritten as $(NpO_2)Cl(H_2O)_{7/4}$, which is close to previously reported $(NpO_2)Cl(H_2O)_2$.[19] In fact, the structures of these two compounds are also closely related. Both structures adopt a 3-D CCI network of neptunyl(V) cations with open channels filled by chloride anions and water molecules, where each NpO_2^+ unit involves four CCIs with neighboring units in a similar square geometry. The main difference between the two structures lies in the bonding at the equatorial plane of NpO_2^+ cations. Compared to **1**, neptunyl(V) pentagonal bipyramids in the dihydrate consist of more H_2O molecules and

less Cl^- anions on average, consistent with the higher water content per Np in the formula. More specifically, half of NpO_2^+ cations in the dihydrate structure are coordinated by three O_w and two O_{yl} atoms and the other half are coordinated by one O_w, two Cl, and two O_{yl} atoms. Furthermore, all chloride anions in the dihydrate are terminal, as such the neptunyl units in the dihydrate are only connected through CCIs. In contrast, some of chloride anions (Cl(3) in Supplementary Figure 9) in **1** are bridging between two Np^V cations, providing additional equatorial connection between neighboring NpO_2^+ units.

$[NMe_4]_2[NpO_2Cl_4]$ (**2**) is isostructural with $[NMe_4]_2[AnO_2Cl_4]$ (An = U, Pu),[1] which crystallizes in the tetragonal space group $P4_2/mnm$. As shown in Supplementary Figure 11, the structure of **2** contains one crystallographically unique nearly linear NpO_2^{2+} cations, each of which is coordinated by four Cl^- anions in the equatorial plane in a tetragonal bipyramidal geometry. The Np−O_{yl} and Np−Cl distances and O_{yl}−Np−O_{yl} angles within these molecular $[NpO_2Cl_4]^{2-}$ anions are 1.765(3) Å, 2.6336(9) and 2.6634(9) Å, and 180.0(2)º, respectively (Supplementary Table 4), which are within the range of those reported for the same dianion associated with other electrolyte cations. For example, the Np−O_{yl} distances within actinyl cations in the structure of $Cs_2[NpO_2Cl_4]$ and $[NBu_4]_2[NpO_2Cl_4]$ are 1.775(17) Å and 1.733(5) Å, respectively.[20, 21] Each discrete $[NpO_2Cl_4]^{2-}$ anion attracts eight neighboring $[NMe_4]^+$ cations through ionic interactions and hydrogen bonding and each $[NMe_4]^+$ cation is connected to four $[NpO_2Cl_4]^{2-}$ anions to form a 3-D network. There is no direct contact between $[NpO_2Cl_4]^{2-}$ moieties with closest Np−Np distances of 8.6031(4) Å.

$[NMe_4]Cl[NpO_2Cl(H_2O)_4]$ (**3**) crystallizes in the triclinic space group $P\bar{1}$. The structure of **1** consists of one crystallographically unique NpO_2^+ cation, two Cl^- anions, four water molecules, and one $[NMe_4]^+$ cation (Supplementary Figure 12). Cation NpO_2^+ is coordinated by one $Cl(2)^-$ anion and four water molecules in the equatorial plane in a pentagonal bipyramidal geometry to form an unprecedented hydrated actinyl mono-chloride complex. Among four coordinating water molecules, three of them (1, 2, 4) have hydrogens sit above and below the equatorial plane, whereas those of the $H_2O(3)$ group sit approximately on the equatorial plane. $H_2O(3)$ participates in a H-bond with a $O_w(1)$ atom within the neptunyl complex with $O_w(3)$−H(33)···$O_w(1)$ distance of 2.794(3) Å. A similar structural arrangement of water molecules within actinyl aquo complexes have been predicted in theoretical studies of $AnO_2(H_2O)_5^{n+}$ (An = U, Np, Pu; n = 1, 2).[22, 23] The Np−O_{yl} distances are 1.838(2) Å and 1.843(2) Å and O−Np−O angles are 176.06(8)º (Supplementary Table 5). The equatorial Np−O_w and Np−Cl(2) distances are in the range of 2.462(2)−2.502(2) Å and 2.8264(7) Å, respectively. The closest distances between the Np center and the $Cl(1)^-$ anion are 4.8393(7) Å, too long to have any significant interactions. Each neutral $[NpO_2Cl(H_2O)_4]$ complex connects to four identical neighbors through water−neptunyl oxygen and water−chloride hydrogen bonding to form 2-D layers on the (010) plane. More specifically, each O_{yl} and Cl(2) atom participates in two and one H-bonds with the water group from surrounding $[NpO_2Cl(H_2O)_4]$ complexes with O_w−H···O_{yl} distances of 2.733(3)−2.859(3) Å and O_w−H···Cl distance of 3.243(2) Å, respectively. The closest Np−Np distances within these layers are 5.5428(4) Å.

As shown in Supplementary Figure 12, single layers of $[NpO_2Cl(2)(H_2O)_4]$ alternates with single neutral layers of $[NMe_4]Cl(1)$ along the [010] direction in the structure of **3**, which are hold together through interlayer H_2O−Cl(1) and NMe_4−Cl(2) hydrogen bonding and $O_{yl}^{\delta-}$/$Cl(2)^-$−NMe_4^+ electrostatic interactions. More specifically, each Cl(1) atom involves in three H-bonds with water groups from neighboring $[NpO_2Cl(H_2O)_4]$ complexes with O_w−H···Cl distance of 3.043(2)−3.128(2) Å. Each Cl(2) atom involves in two H-bonds with the neighboring methyl groups with C−H···Cl distances of 3.872(3) and 3.866(3) Å. In addition, $Cl^-(2)$ anion is surrounded by four NMe_4^+ cations with the Cl−N distances of 4.184(2)−5.156(2) Å, which are close or shorter than those for contact NMe_4^+−Cl^- ion pairs (5.0 Å) in

solution.[24] Each O_{yl} has one close neighboring NMe_4^+ cation with the O_{yl}−N distances of 4.062(3) Å and closest O_{yl}−C distances of 3.418(4) Å for O_{yl}(1) and O_{yl}−N distances of 4.660(3) Å and closest O_{yl}−C distances of 3.418(4) Å for O_{yl}(2). Alternatively, the structure of **3** can be described as alternating cationic layers of $[NpO_2(H_2O)_4]^+$ and NMe_4^+, which are separated by layers of Cl^- anions. Similar to $Cl(2)^-$, each $Cl(1)^-$ anion is surrounded by four nearby NMe_4^+ cations with Cl−N distances of 4.183(2)−5.016(2) Å. Each Cl(1) atom involves in two H-bonds with the neighboring methyl groups with C−H···Cl distances of 3.744(3) and 3.833(3) Å. The closest distances between the centers of two cationic species, Np−N, across these layers are 5.292(4) Å.

Raman Spectroscopy. Neptunyl (NpO_2^{n+}) cations exhibit three fundamental Np–O_{yl} vibrational modes, including ν_1 (symmetric stretching, Raman active), ν_2 (doubly degenerate bending, infrared active), and ν_3 (asymmetric stretching, infrared active). These vibrational modes are sensitive to the neptunyl coordination environment, which includes the inner–sphere coordination of ligands in the equatorial plane or the outer–sphere coordination of other species to the yl–O atoms. In particular, the formation of CCI complexes, in which the yl–O atom of one neptunyl cation is coordinated within the inner–sphere equatorial plane of a second neptunyl cation, can make both neptunyl stretching modes Raman active and can shift the stretching bands toward lower frequencies by changing the site symmetry of the neptunyl units and Np−O_{yl} interactions.[25, 26] The ν_1 and ν_3 modes of NpO_2^+ units in solutions and solids have been observed in the region of 630−770 cm^{-1} and 770−850 cm^{-1}, respectively.[25–30] In comparison, the ν_1 of NpO_2^{2+} units have been observed in the region of 802−863 cm^{-1} in several Raman spectra of neptunyl(VI) aqueous solutions and solid compounds without CCIs.[28, 30, 31] The bending bands of neptunyl cations are less certain due to their much lower intensity and potential overlap with ligand modes.

Raman spectra of **1**−**3** and select green amorphous products (**X**) from Np^V/LiCl reactions in the region of 600−900 cm^{-1} are presented in Supplementary Figure 13. At first glance, the Raman spectra of **1** and **X** are more complex than those of **2** and **3** that lack CCIs as expected. Most of the vibrational bands of **1** and **X** in the regions of neptunyl stretching modes are comparable to those observed for green products from evaporating acidic Np^V chloride solutions including $(NpO_2)Cl(H_2O)_2$, mixed-valent $Na_xNp^{IV}(Np^VO_2)_6(OH)_{1+x}Cl_9(H_2O)_{8-x}$ ($0<x\leq1$) and other unidentified green phases.[18, 26] More specifically, Raman bands of **1** located at 672, 708 (vw = very weak), 744 (vw), 806 (w = weak), and 847 (w) cm^{-1} match with most of bands for $(NpO_2)Cl(H_2O)_2$ (675, 800 (w), and 846 (w) cm^{-1}) and other unidentified green products (671/672, 744 (vw), 805 (w), 825 (w), and 846 (w) cm^{-1}) from slow evaporation reactions of Np^V/HCl.[26] This is consistent with X-ray structural analyses, which reveal comparable Np−O_N distances and similar CCI connectivities for **1** and $(NpO_2)Cl(H_2O)_2$.[26, 32] Raman spectra of **X** depend on the measured samples, which suggests the formation of a mixture of Np containing products. Most of Raman bands of **X** resemble those of green products from rapid evaporation reactions of Np^V/HCl.[26] For example, broad bands in the 680−730 cm^{-1} and 780−820 cm^{-1} regions are similar to those of $Na_xNp^{IV}(Np^VO_2)_6(OH)_{1+x}Cl_9(H_2O)_{8-x}$ ($0<x\leq1$).[26] The strong vibration band located at 633 cm^{-1} is close to the lowest reported frequency (635 cm^{-1}) of the ν_1 modes of NpO_2^+ units observed for $NaNpO_2(OH)_2$.[33] A similar strong band located at 624 cm^{-1} has been observed for some of green products from rapid evaporation reactions of Np^V/HCl.[26]

The intense peak at 797 cm^{-1} in the spectrum of **2** and 741 cm^{-1} in the spectrum of **3** can be assigned to the ν_1 mode of O=Np=O moieties, whereas the weak bands at 754 cm^{-1} in the spectrum of **2** and 757 cm^{-1} in the spectrum of **3** can be assigned to the ν_1 mode of NMe_4^+ units. The ν_1 frequency of neptunyl(VI) cations in compound **2** is comparable to the value observed for $Cs_2[NpO_2Cl_4]$ (802 cm^{-1}) and $[NBu_4]_2[NpO_2Cl_4]$ (800 cm^{-1}).[20, 21] The ν_1 band of neptunyl(V) cations in compound **3** shifts to a lower frequency compared to

typical values observed for those that do not involve in CCIs and is close to those for CCI dimers in solution (738 cm^{-1}). For example, the ν_1 band of discrete NpO_2^+ cations has been observed at ca. 773 cm^{-1} in the Raman spectra of $Na_3(NpO_2)(SeO_4)_2(H_2O)$.[32] This is consistent with the trend of Np−O_{yl} distances observed in the structures of **3**, $Na_3(NpO_2)(SeO_4)_2(H_2O)$, and Np(V) CCI compounds.

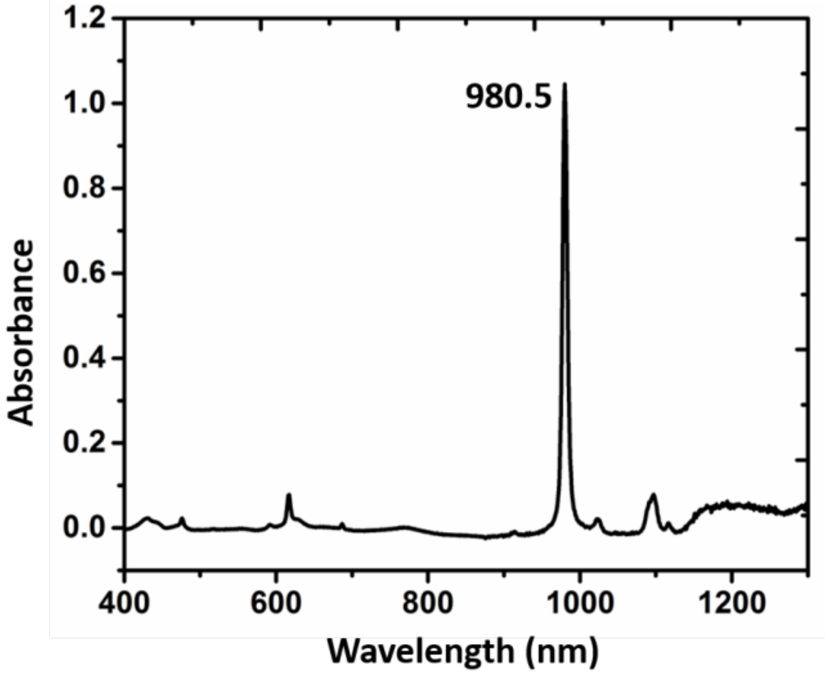

Supplementary Figure 1. Visible-Near-Infrared absorption spectrum of a diluted aliquot of the 0.1 M Np^{V} (in ~1 M HCl) stock solution.

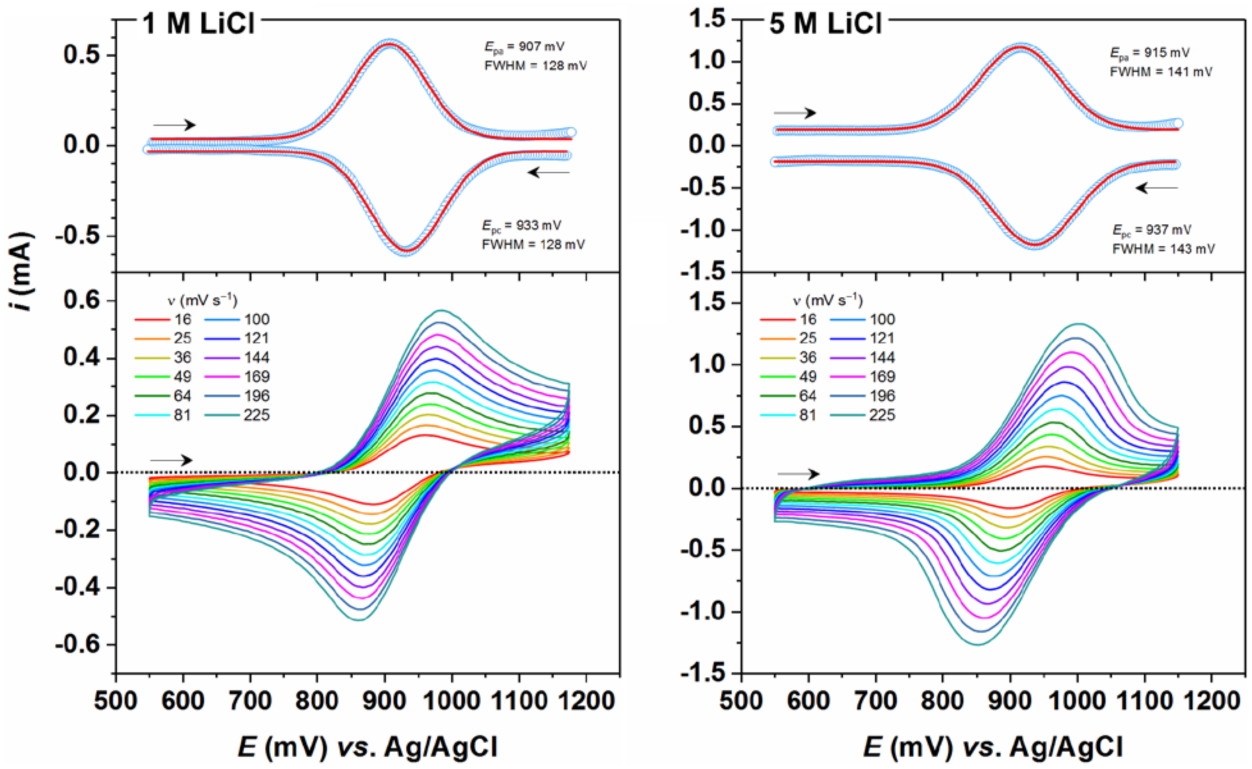

Supplementary Figure 2. Voltammetry data for solutions of 5 mM Np^V dissolved in 1 M LiCl (left panels) or 5 M LiCl (right panels). Experimental DPV data (blue circles, top) were collected at ν = 20 mV s^{-1} from separate anodic ($E_{initial}$ = 550 mV to E_{final} = 1175/1150 mV) and cathodic ($E_{initial}$ = 1175/1150 mV to E_{final} = 550 mV) scans. Peak potentials (E_{pa}, E_{pc}) and corresponding full-width-half-maxima (FWHM) were determined from separate Gaussian fits (red lines, top, Origin2016) of each DPV scan. For CV data (bottom), the initial and cathodic switching potential ($E_{\lambda c}$) = 550 mV, and the anodic switching potential ($E_{\lambda a}$) = 1175/1150 mV. Arrows indicate initial scan direction for DPV and CV data. The background DPV and CV data for Np-free supporting electrolyte solutions (not shown for clarity) were featureless.

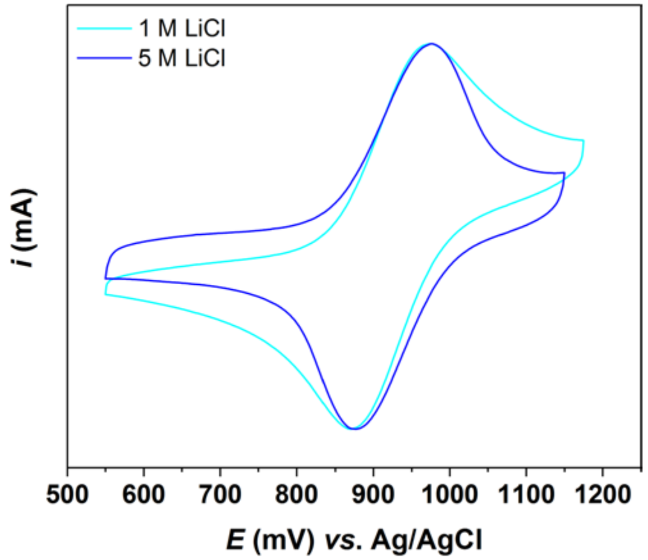

Supplementary Figure 3. Current normalized cyclic voltammetry data for solutions of 5 mM Np^{V} dissolved in 1 M LiCl or 5 M LiCl. The initial and cathodic switching potential ($E_{\lambda c}$) = 550 mV, and the anodic switching potential ($E_{\lambda a}$) = 1175/1150 mV; v = 100 mV s^{-1}.

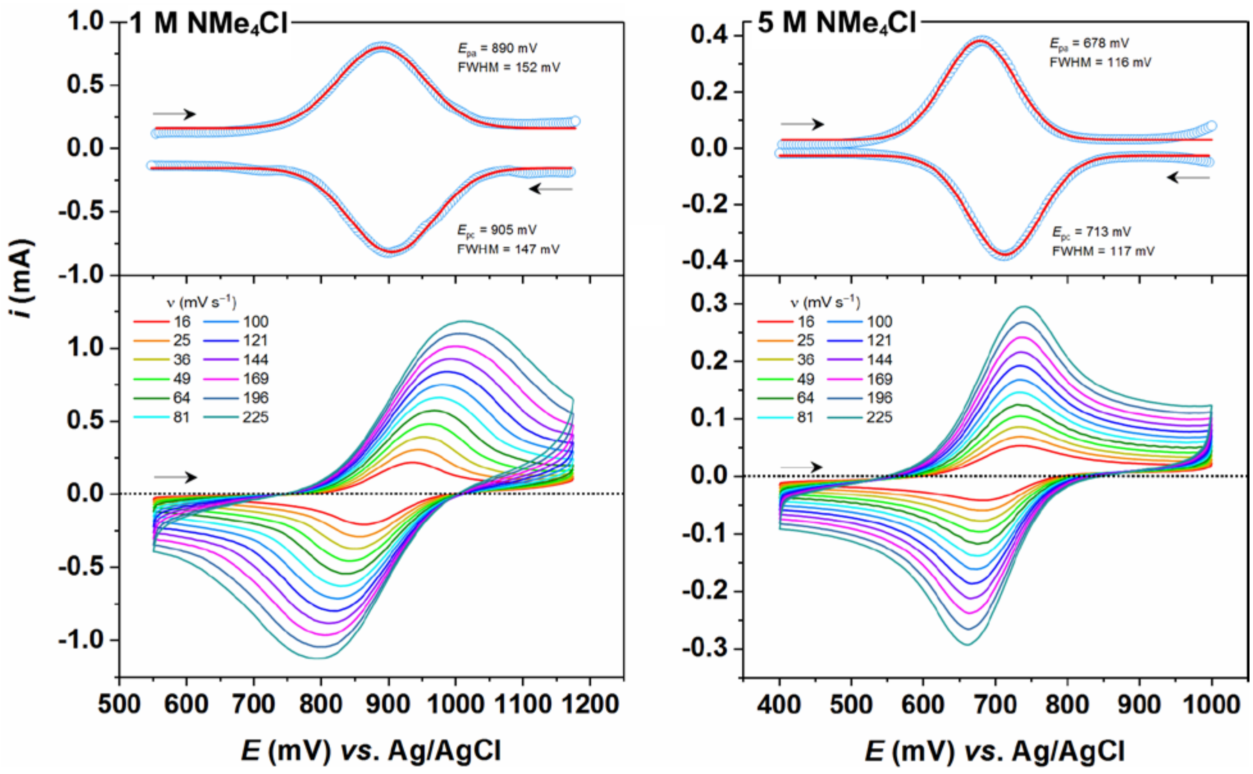

Supplementary Figure 4. Voltammetry data for solutions of 5 mM Np^{V} dissolved in 1 M NMe_4Cl (left panels) or 5 M NMe_4Cl (right panels). Experimental DPV data (blue circles, top) were collected at ν = 20 mV s^{-1} from separate anodic (E_{initial} = 550/400 mV to E_{final} = 1750/1000 mV) and cathodic (E_{initial} = 1175/1000 mV to E_{final} = 550/400 mV) scans. Peak potentials (E_{pa}, E_{pc}) and corresponding full-width-half-maxima (FWHM) were determined from separate Gaussian fits (red lines, top, Origin2016) of each DPV scan. For CV data (bottom), the initial and cathodic switching potential ($E_{\lambda c}$) = 550/400 mV, and the anodic switching potential ($E_{\lambda a}$) = 1175/1000 mV. Arrows indicate initial scan direction for DPV and CV data. The background DPV and CV data for Np-free supporting electrolyte solutions (not shown for clarity) were featureless.

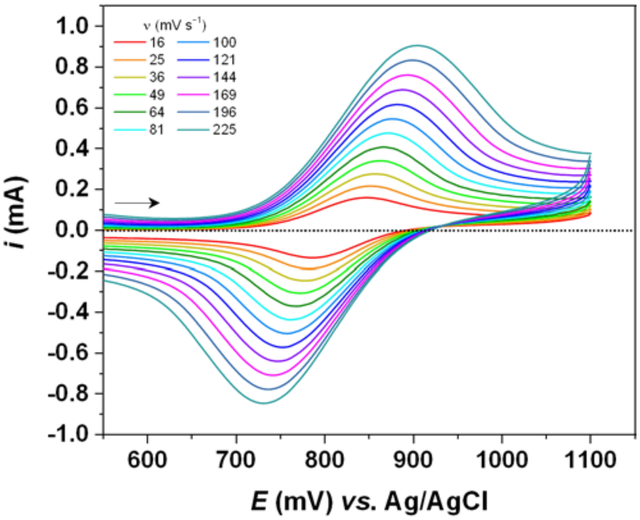

Supplementary Figure 5. Cyclic voltammetry data for 5 mM Np^{V} dissolved in 3 M NEt_4Cl. The initial and cathodic switching potential ($E_{\lambda c}$) = 550 mV, and the anodic switching potential ($E_{\lambda a}$) = 1150 mV; the arrow indicates initial scan direction. The background CV data for Np-free supporting electrolyte solutions (not shown for clarity) were featureless.

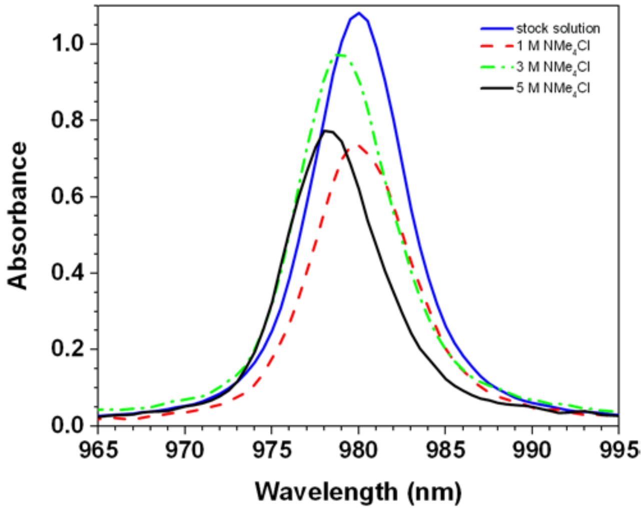

Supplementary Figure 6. Vis-NIR absorption spectra for a diluted aliquot of the 0.1 M Np^{V} stock solution (see Supplementary Figure 1) and solutions of ≈5 mM Np^{V} dissolved in 1 M, 3 M, and 5 M NMe_4Cl. Only the spectral range illustrating changes in the 980.2 nm line is shown. Molar absorptivities range from approximately 390 L M^{-1} cm^{-1} to 420 L M^{-1} cm^{-1}.

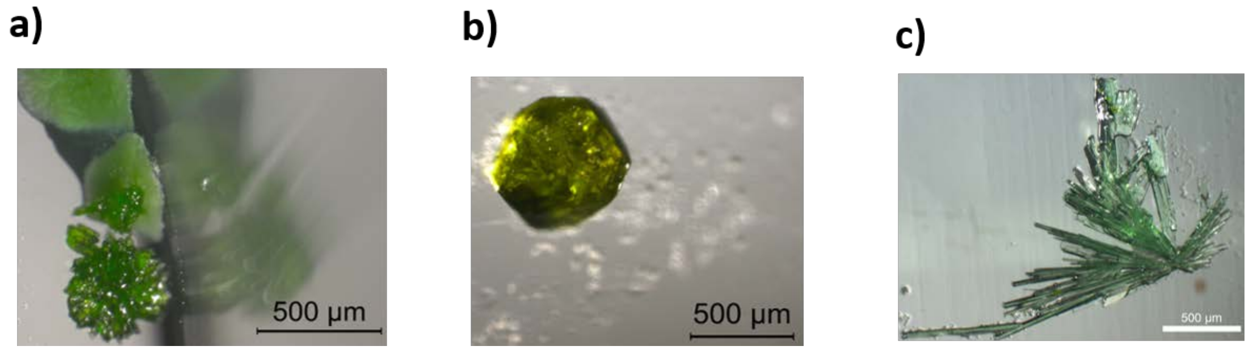

Supplementary Figure 7. Images of solid products from evaporation reactions for a) $(NpO_2)_4Cl_4(H_2O)_7$ (**1**), green crystals; b) $[NMe_4]_2[NpO_2Cl_4]$ (**2**), yellow-green crystals; c) $[NMe_4]Cl[NpO_2Cl(H_2O)_4]$ (**3**), teal crystals.

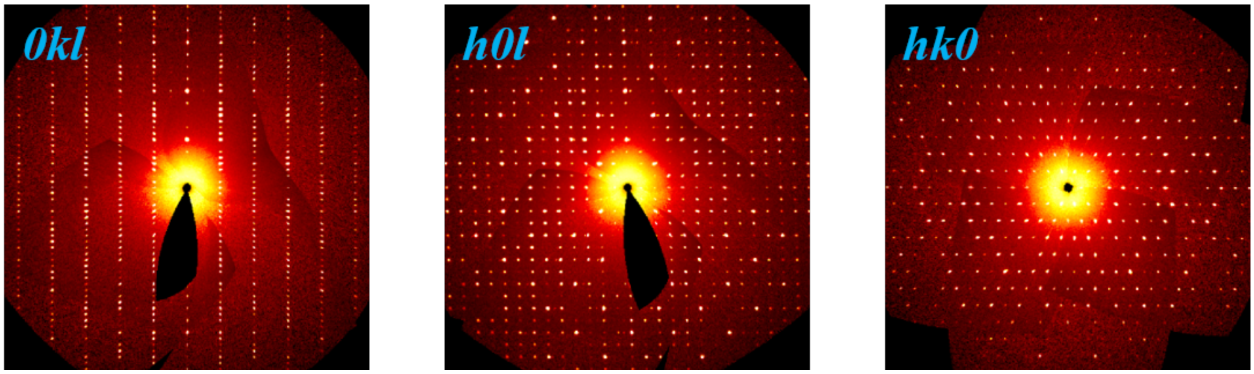

Supplementary Figure 8. Synthesized *hk1* precession images simulated in APEX2 from diffraction frames of a crystal of $(NpO_2)_4Cl_4(H_2O)_7$ (**1**).

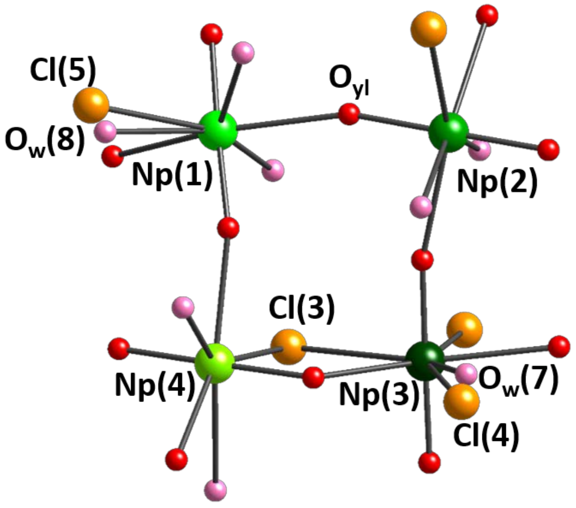

Supplementary Figure 9. Local coordination environments of Np atoms (green sphere) surrounded by O_{yl} (yl = actinyl, red sphere), O_w (w = water, pink sphere), and Cl (orange sphere) atoms in the structure of $(NpO_2)_4Cl_4(H_2O)_7$ (**1**). $Cl^{-}(4)$ and $Cl^{-}(5)$ anions are disordered with $H_2O_w(7)$ and $H_2O_w(8)$ molecules, respectively.

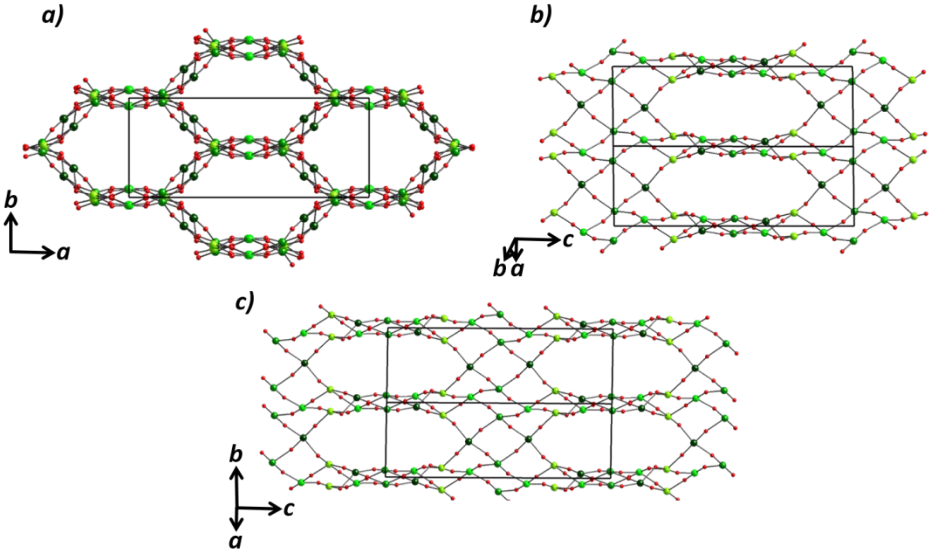

Supplementary Figure 10. A three-dimensional network of cation−cation bound neptunyl square nets with open channels along the [001] (a), [1−10] (b), and [110] (c) directions in the structure of $(NpO_2)_4Cl_4(H_2O)_7$ (**1**). Chloride anions and water molecules that reside inside of channels are omitted from the figure for clarity.

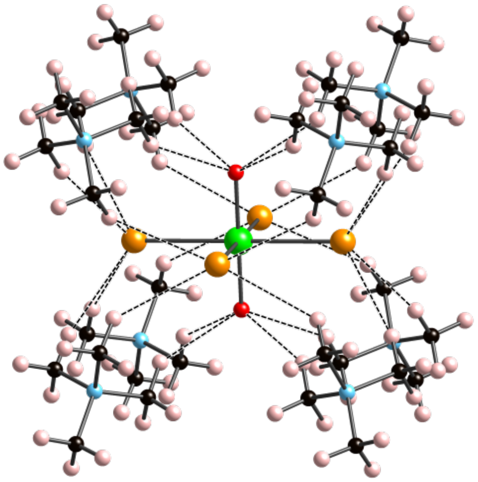

Supplementary Figure 11. The connectivities between molecular $[NpO_2Cl_4]^{2-}$ anions and $[NMe_4]^+$ cations in the structure of $[NMe_4]_2[NpO_2Cl_4]$ (**2**). H-bonding is shown with dashed lines. Green, red, orange, blue, black, and beige spheres represent Np, O_{yl}, Cl, N, C, and H atoms, respectively.

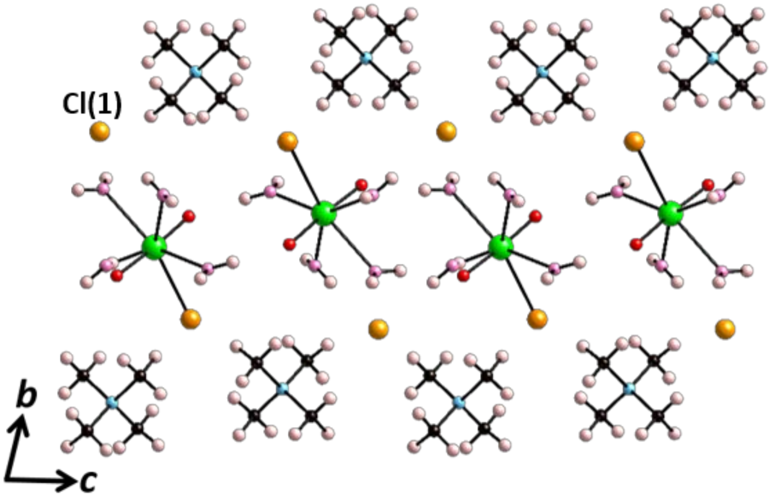

Supplementary Figure 12. The overall packing of $[NpO_2Cl(H_2O)_4]$ complexes, $[NMe_4]^+$ cations, and $Cl(1)^-$ anions in the structure of $NMe_4]Cl[NpO_2Cl(H_2O)_4]$ (**3**). Green, red, pink, orange, blue, black, and beige spheres represent Np, O_{yl}, O_w, Cl, N, C, and H atoms, respectively. H-bonding is omitted for clarity.

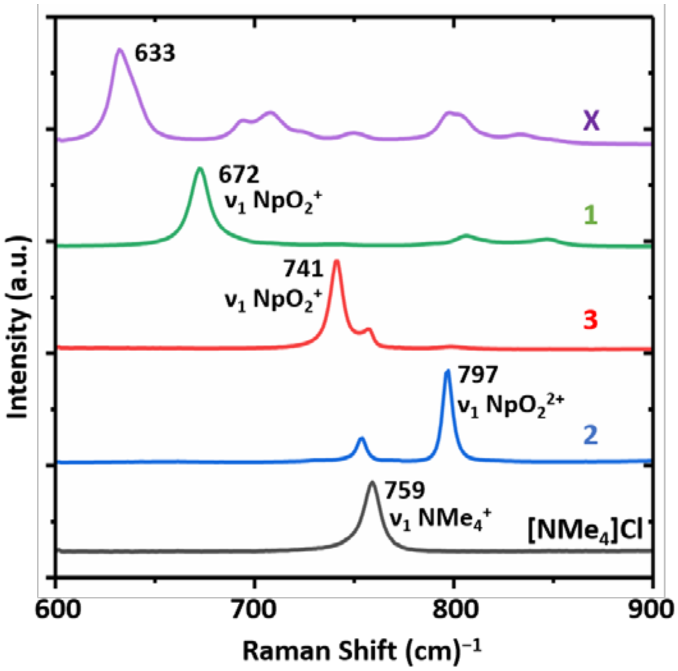

Supplementary Figure 13. Raman spectra of selected green amorphous products (X) from Np^{V}/LiCl reactions (purple), $(NpO_2)_4Cl_4(H_2O)_7$ (**1**) (green), $[NMe_4]_2[NpO_2Cl_4]$ (**2**) (blue), and $[NMe_4]Cl[NpO_2Cl(H_2O)_4]$ (**3**) (red). A Raman spectrum of $[NMe_4]Cl$ (black) is included for comparison.[19] The intensities for all spectra are scaled to facilitate comparison.

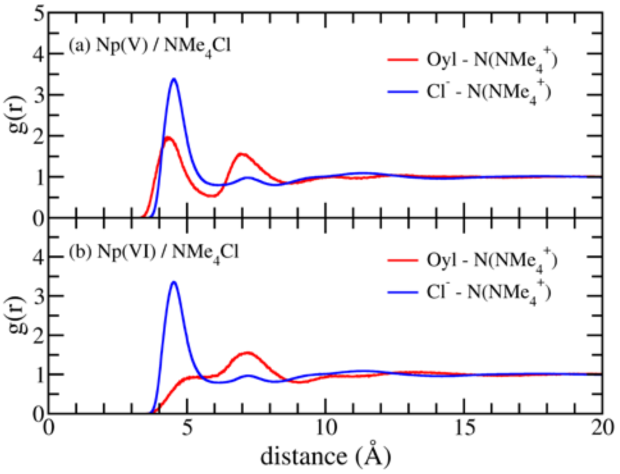

Supplementary Figure 14. Radial distribution functions between O_{yl}–$[NMe_4]^+$ and between Cl^-–$[NMe_4]^+$ for the two systems of Np^V and Np^{VI}, both with 5M NMe_4Cl.

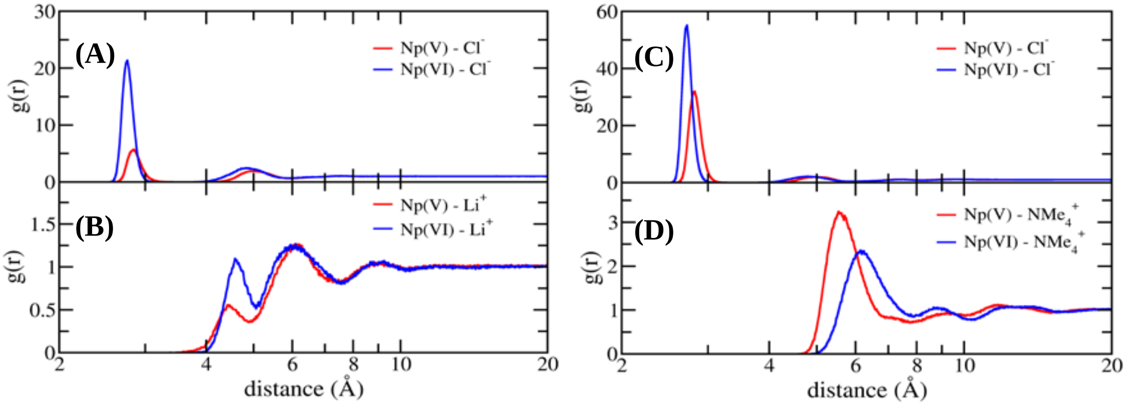

Supplementary Figure 15. Radial distribution function between (A) Np–Cl^- and (B) Np–Li^+ for both Np^{V} and Np^{VI} in the 5 M LiCl system. (C, D) Corresponding plots in the 5 M NMe_4Cl system.

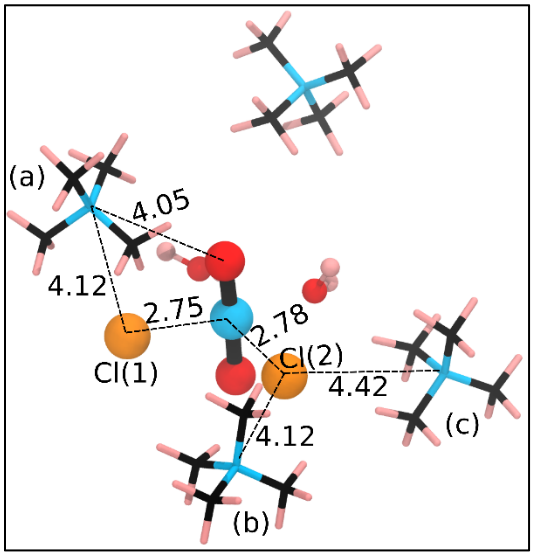

Supplementary Figure 16. Atomistic simulation snapshot showing the local coordination around one $Np^{V}O_2^{+}$ ion in the 5 M NMe_4Cl solution. Dashed lines are just to guide the eyes in terms of distances (in the unit of Å). One $[NMe_4]^+$ (a) is interacting with one Cl^- [Cl(1), 4.12 Å] and one O_{yl} (4.05 Å), which is similar to the structure of compound **3**. Additionally, two $[NMe_4]^+$ ions (b, c) are interacting with the $Np^{V}O_2^{+}$ ion bridged by one Cl^- ion [Cl(2)].

Supplementary Table 1. Electrode potentials for the Np^{VI}/Np^{V} couple in various supporting electrolytes.

solution conditions[a]	*E* (V) *vs.* Ag/AgCl	ref.
1 M LiCl	+0.924(2)[b]	this work
5 M LiCl	+0.929(3)[b]	this work
1 M NMe_4Cl	+0.903(4)[b]	this work
3 M NMe_4Cl	+0.818(7)[b]	this work
5 M NMe_4Cl	+0.704(7)[b]	this work
3 M NEt_4Cl	+0.822(7)[b]	this work
1 M NMe_4Cl / 4 M LiCl	+0.905(2)[b]	this work
3 M NMe_4Cl / 2 M LiCl	+0.851(2)[b]	this work
1 M $HClO_4$ [40 mM Np^{VI}]	+0.90	[34]
1 M HNO_3 / 4.0 M NH_4NO_3 [40 mM Np^{VI}]	+0.91	[34]
4.4 M HNO_3 / 6.0 M NH_4NO_3 [40 mM Np^{VI}]	+0.89	[34]
1 M $HClO_4$	+0.931(15)	[35]
1.5 M Na_2CO_3, pH 11.8 [10 mM Np^{VI}]	+0.25[c]	[36]
standard electrode potential	+0.959(4)	[37]

[a]Unless listed in brackets, Np was present initially as Np^{V} at a total concentration of 5 mM.
[b]Half-wave potential ($E_{1/2}$), averaged over all measured scan rates, with 2σ uncertainty given in parentheses; $E_{1/2} \approx E°$.
[c]Neither $E_{1/2}$ nor $E°$ values were reported; we graphically estimated this value based on cyclic voltammograms shown in Figure 3 of the cited reference. Neptunium(V) speciation under these conditions was reported as $[NpO_2(CO_3)_3]^{5-}$.

Supplementary Table 2. Crystal data and structure refinements for $(NpO_2)_4Cl_4(H_2O)_7$ (**1**), $[NMe_4]_2[NpO_2Cl_4]$ (**2**), and $[NMe_4]Cl[NpO_2Cl(H_2O)_4]$ (**3**).

	$(NpO_2)_4Cl_4(H_2O)_7$	$[NMe_4]_2[NpO_2Cl_4]$	$[NMe_4]Cl[NpO_2Cl(H_2O)_4]$
Fw	1343.91	559.09	486.11
Color and habit	Green prism	Yellow-green block	Teal needle
Crystal system	Monoclinic	Tetragonal	Triclinic
Space group	$C2/c$	$P4_2/mnm$	$P\bar{1}$
a, Å	20.2198(9)	9.1132(6)	5.5929(3)
b, Å	8.3551(3)	9.1132(6)	11.0308(7)
c, Å	23.2897(9)	11.3998(7)	11.1054(7)
α, °	90	90	75.4409(8)
β, °	91.720(1)	90	86.6656(9)
γ, °	90	90	80.9676(9)
V, $Å^3$	3932.7(3)	946.8(1)	654.79(7)
Z	8	2	2
ρ_c, g/cm^3	4.54	1.96	2.47
μ, mm^{-1}	21.56	6.05	8.35
F(000)	4592	526	452
Crystal size, mm^3	0.050 x 0.040 x 0.030	0.054 x 0.038 x 0.028	0.076 x 0.026 x 0.006
Theta range for data collection, °	1.750 to 33.231	2.862 to 33.181	1.895 to 33.073
Index ranges	$-30 \le h \le 29$, $-12 \le k \le 12$, $-35 \le l \le 35$	$-13 \le h \le 13$, $-13 \le k \le 13$, $17 \le l \le 17$	$-8 \le h \le 8$, $-15 \le k \le 16$, $0 \le l \le 16$
Total reflections collected	34235	15957	4525
Independent reflections [$I > 2\sigma(I)$]	6189 [R_{int} = 0.0314]	810 [R_{int} = 0.0296]	4363 [R_{int} = 0.0204]
Data/ restraints/ parameters	7208 / 0 / 226	998/ 0 / 39	4525 / 12 / 156
Goodness-of-fit on F^2	1.055	1.122	1.052
Final R indices [$I > 2\sigma(I)$]	R_1 = 0.0334, wR^2 = 0.0767	R_1 = 0.0158, wR^2 = 0.0361	R_1 = 0.0187, wR^2 = 0.0415
R indices (all data)	R_1 = 0.0411, wR^2 = 0.0794	R_1 = 0.0219, wR^2 = 0.0382	R_1 = 0.0201, wR^2 = 0.0419
Largest diff. peak and hole, e.$Å^{-3}$	4.381 and -3.171	1.866 and -0.281	1.853 and -0.841

[a]For all structures, T = 100(2) K, λ = 0.71073 Å, Completeness to theta = 25.242°: 100%, Extinction coefficient: n/a, Refinement method: Full-matrix least-squares on F^2.

Supplementary Table 3. Selected interatomic distances (Å) and angles (º) for $(NpO_2)_4Cl_4(H_2O)_7$ (**1**).

Np(1)−O(1)	1.852(5)	Np(2)−Cl(1)	2.799(2)	Np(4)−O_w(5)	2.534(6)
Np(1)−O(2)	1.838(5)	Np(3)−O(5)	1.836(5)	Np(4)−Cl(3)	2.783(2)
Np(1)−O(3)	2.385(5)	Np(3)−O(6)	1.842(5)	Np(1)−Np(2)	4.1586(4)
Np(1)−O(7)	2.397(5)	Np(3)−O(4)	2.404(5)	Np(1)−Np(2)	4.1786(4)
Np(1)−O_w(1)	2.607(9)	Np(3)−O(8)	2.466(5)	Np(1)−Np(4)	4.2058(4)
Np(1)−O_w(6)	2.440(7)	Np(3)−O_w(7)	2.35(2)	Np(1)−Np(4)	4.2076(4)
Np(1)−O_w(8)	2.52(2)	Np(3)−Cl(2)	2.765(2)	Np(2)−Np(3)	4.1895(4)
Np(1)−Cl(5)	2.828(6)	Np(3)−Cl(3)	2.892(2)	Np(2)−Np(3)	4.2087(4)
Np(2)−O(3)	1.845(5)	Np(3)−Cl(4)	2.795(5)	Np(3)−Np(4)	3.8589(4)
Np(2)−O(4)	1.846(5)	Np(4)−O(7)	1.842(5)	Np(3)−Np(4)	4.2454(3)
Np(2)−O(2)	2.387(5)	Np(4)−O(8)	1.853(5)	O(1)−Np(1)−O(2)	176.7(3)
Np(2)−O(5)	2.404(5)	Np(4)−O(1)	2.425(5)	O(3)−Np(2)−O(4)	178.9(3)
Np(2)−O_w(2)	2.537(7)	Np(4)−O(6)	2.410(5)	O(5)−Np(3)−O(6)	179.1(2)
Np(2)−O_w(4)	2.508(6)	Np(4)−O_w(3)	2.511(6)	O(7)−Np(2)−O(8)	176.4(2)

Supplementary Table 4. Selected interatomic distances (Å) and angles (º) for $[NMe_4]_2[NpO_2Cl_4]$ (**2**).

Np(1)−O(1) x 2	1.765(3)	C−H···O(1)	3.385(3)
Np(1)−Cl(1) x 2	2.6634(9)	O(1)−N(1) x 4	4.5417(9)
Np(1)−Cl(1) x 2	2.6336(9)	C−H···Cl(1) x 4	3.901(3)
Np(1)−C(1) x 8	4.578(2)	Cl(1)−N(1) x 4	4.3377(2)
Np(1)−C(1) x 8	5.114(3)	C−H···Cl(2) x 4	3.686(2)
Np(1)−N(1) x 8	5.3745(3)	Cl(2)−N(1) x 4	4.5617(3)
Np(1)−Np(1)	8.6031(4)	O(1)−Np(1)−O(1)	180.0(2)

Supplementary Table 5. Selected interatomic distances (Å) and angles (º) for $[NMe_4]Cl[NpO_2Cl(H_2O)_4]$ (**3**).

Np(1)−O(1)	1.838(2)	O_w(3)−H(33)···Cl(1)	3.090(2)
Np(1)−O(2)	1.843(2)	O_w(4)−H(44)···Cl(1)	3.128(2)
Np(1)−O_w(1)	2.478(2)	C(1)−H(1A)···Cl(1)	3.744(3)
Np(1)−O_w(2)	2.492(2)	C(1)−H(1C)···Cl(1)	3.833(3)
Np(1)−O_w(3)	2.502(2)	Cl(1)−C(1)	3.310(3)
Np(1)−O_w(4)	2.462(2)	Cl(1)−N(1)	4.183(2)
Np(1)−Cl(2)	2.8264(7)	Cl(1)−N(1)	4.190(2)
Np(1)−C(4)	4.625(3)	Cl(1)−N(1)	4.767(2)
Np(1)−Cl(1)	4.8393(7)	Cl(1)−N(1)	5.016(2)
Np(1)−N(1)	5.292(4)	O_w(2)−H(2)···Cl(2)	3.243(2)
Np(1)−Np(1)	5.5428(4)	C(3)−H(3A)···Cl(2)	3.872(3)
O_w(1)−H(1)···O(1)	2.832(3)	C(4)−H(4A)···Cl(2)	3.866(3)
O_w(4)−H(4)···O(1)	2.756(3)	Cl(2)−C(4)	3.381(3)
O(1)−C(1)	3.418(4)	Cl(2)−N(1)	4.184(2)
O(1)−N(1)	4.062(3)	Cl(2)−N(1)	4.381(2)
O_w(2)−H(22)···O(2)	2.733(3)	Cl(2)−N(1)	4.853(3)
O_w(3)−H(3)···O(2)	2.859(3)	Cl(2)−N(1)	5.156(2)
O(2)−C(3)	3.937(4)	O_w(3)−H(33)···O_w(1)	2.794(3)
O(2)−N(1)	4.660(3)	O(1)−Np(2)−O(5)	176.06(8)
O_w(1)−H(11)···Cl(1)	3.043(2)		

Supplementary Table 6. Compositions of the systems investigated in the MD Simulations.[a]

system	NpO_2^{n+} [a]	Li^{+} [b]	NMe_4^{+} [c]	H_3O^{+} [d]	Cl^{-}	water
Np(VI), 5M LiCl	30	1500	-	3	1563	15126
Np(VI), 5M NMe_4Cl	30	-	1500	3	1563	9988
Np(V), 5M LiCl	30	1500	-	3	1533	15126
Np(V), 5M NMe_4Cl	30	-	1500	3	1533	9988

a) [NpO_2^{n+} * Cl_n]= 0.1 M with n = 1, 2.
b) [Li * Cl] = 5 M.
c) [NMe_4 * Cl] = 5 M.
d) [H_3O * Cl] = 0.01 M (pH=2)

Supplementary Table 7. Average calculated (MD simulations) and experimental distances (Å) of O_{yl}–$[NMe_4]^+$ and Cl^-–$[NMe_4]^+$.

	Np^{V} (5 M NMe_4Cl)		Np^{VI} (5 M NMe_4Cl)	
	Simulation[a]	Experiment[b]	Simulation[a]	Experiment[c]
O_{yl}–$[NMe_4]^+$	4.28	4.062	5.10	4.5417
Cl^-–$[NMe_4]^+$	4.54	4.381	4.52	4.3377

(a) see Supplementary Figure 15;
(b) from compound **3**;
(c) from compound **2**.

Supplementary Table 8. Thermodynamic calculations.

	reaction			E (V) *vs*. SHE	log β	ΔG (kJ mol^{-1})[a]
	$Np^{VI}O_2^{2+} + e^-$	$\rightarrow$	$Np^{V}O_2^{+}$	+0.93[b]	--	–89.73
+	$Np^{VI}O_2Cl^{+}$	$\rightarrow$	$Np^{VI}O_2^{2+} + Cl^-$	--	–0.40[c]	+2.28
+	$Np^{V}O_2^{+} + Cl^-$	$\rightarrow$	$Np^{V}O_2Cl$	--	–0.29[d]	+1.66
=	$Np^{VI}O_2Cl^{+} + e^-$	$\rightarrow$	$Np^{V}O_2Cl$	+0.89[e]	--	–85.79[f]
	$\Delta\Delta G = \Delta G(Np^{VI}O_2^{2+}/Np^{V}O_2^{+}) - \Delta G(Np^{VI}O_2Cl^{+}/Np^{V}O_2Cl) = -3.94$					
	$Np^{VI} + e^-$	$\rightarrow$	Np^{V} (in 5 M NMe_4Cl)	+0.70[b]	--	–67.54
	$\Delta\Delta G = \Delta G(Np^{VI}O_2^{2+}/Np^{V}O_2^{+}) - \Delta G(Np^{VI}/Np^{V}$, 5 M $NMe_4Cl) = -22.19$					

[a]Unless otherwise noted, ΔG values were calculated using $\Delta G = -nFE$ or $\Delta G = -RT \cdot ln\beta$.
[b]This work.
[c]Guillaumont et al.[38]
[d]Neck et al.[39]
[e]Calculated from ΔG.
[f]Calculated as the sum of ΔG for three reactions shown.

Supplementary References.

1. Schnaars, D. D.; Wilson, R. E. *Inorg. Chem.* **52**, 14138-14147 (2013).
2. Gelato, L. M.; Parthe, E. *J. Appl. Crystallogr.* **20**, 139-143 (1987).
3. Hess, B.; Kutzner, C.; van der Spoel, D.; Lindahl, E. *J. Chem. Theory Comput.* **4**, 435-447 (2008).
4. Pomogaev, V.; Tiwari, S. P.; Rai, N.; Goff, G. S.; Runde, W.; Schneider, W. F.; Maginn, E. J. *Phys. Chem. Chem. Phys.***15**, 15954-15963 (2013).
5. Miyamoto, S.; Kollman, P. A. *J. Comput. Chem.* **13**, 952-962 (1992).
6. van Keulen, S. C.; Gianti, E.; Carnevale, V.; Klein, M. L.; Rothlisberger, U.; Delemotte, L. *J. Phys. Chem. B* **121**, 3340-3351 (2017).
7. Klauda, J. B.; Venable, R. M.; Freites, J. A.; OConnor, J. W.; Tobias, D. J.; Mondragon-Ramirez, C.; Vorobyov, I.; MacKerell, A. D.; Pastor, R. W. *J. Phys. Chem. B* **114**, 7830-7843 (2010).
8. Huang, J.; Rauscher, S.; Nawrocki, G.; Ran, T.; Feig, M.; de Groot, B. L.; Grubmuller, H.; MacKerell Jr, A. D. *Nat. Methods* **14**, 71-73 (2017).
9. Martínez, L.; Andrade, R.; Birgin, E. G.; Martínez, J. M. *J. Comput. Chem.* **30**, 2157-2164 (2009).
10. Darden, T.; York, D.; Pedersen, L. *J. Chem. Phys.* **98**, 10089-10092 (1993).
11. Essmann, U.; Perera, L.; Berkowitz, M. L.; Darden, T.; Lee, H.; Pedersen, L. *J. Chem. Phys.* **103**, 8577-93 (1995).
12. Lee, J.; Cheng, X.; Swails, J. M.; Yeom, M. S.; Eastman, P. K.; Lemkul, J. A.; Wei, S.; Buckner, J.; Jeong, J. C.; Qi, Y.; Jo, S.; Pande, V. S.; Case, D. A.; Brooks, C. L.; MacKerell, A. D.; Klauda, J. B.; Im, W. *J. Chem. Theory Comput.* **12**, 405-413 (2016).
13. Hess, B. *J. Chem. Theory Comput.* **4**, 116-122 (2008).
14. Matsika, S.; Pitzer, R. M.; Reed, D. T. *J. Phys. Chem. A* **104**, 11983-11992 (2000).
15. Sullivan, J. C.; Hindman, J. C.; Zielen, A. J. *J. Am. Chem. Soc.* **83**, 3373-3378 (1961).
16. Xian, L.; Tian, G.; Zheng, W.; Rao, L. *Dalton Trans.* **41**, 8532-8538 (2012).
17. Freiderich, J. W.; Burn, A. G.; Martin, L. R.; Nash, K. L.; Clark, A. E. *Inorg. Chem.* **56**, 4788-4795 (2017).
18. Jin, G. B. *Inorg. Chem.* **55**, 2612-2619 (2016).
19. Grigorev, M. S.; Bessonov, A. A.; Krot, N. N.; Yanovskii, A. I.; Struchkov, Y. T. *Radiochemistry* **35**, 382-387 (1993).
20. Wilkerson, M. P.; Arrington, C. A.; Berg, J. M.; Scott, B. L. *J. Alloys Compd.* **444**, 634-639 (2007).
21. Wilkerson, M. P.; Dewey, H. J.; Gordon, P. L.; Scott, B. L. *J. Chem. Crystallogr.* **34**, 807-811 (2004).
22. Cao, Z.; Balasubramanian, K. *J. Chem. Phys.* **123**, 114309 (2005).

23. Yin, Y.-P.; Dong, C.-Z.; Ding, X.-B. *J. Phys. Chem. A* **119**, 3253-3260 (2015).

24. Buckner, J. K.; Jorgensen, W. L. *J. Am. Chem. Soc.* **111**, 2507-2516 (1989).

25. Krot, N. N.; Grigoriev, M. S. *Russ. Chem. Rev.* **73**, 89-100 (2004).

26. Jin, G. B. *Inorg. Chem.* **52**, 12317-12319 (2013).

27. Jones, L. H.; Penneman, R. A. *J. Chem. Phys.* **21**, 542-544544 (1953).

28. Basile, L. J.; Sullivan, J. C.; Ferraro, J. R.; LaBonville, P. *Appl. Spectrosc.* **28**, 142-145 (1974).

29. Guillaume, B.; Begun, G. M.; Hahn, R. L. *Inorg. Chem.* **21**, 1159-1166 (1982).

30. Madic, C.; Hobart, D. E.; Begun, G. M. *Inorg. Chem.* **22**, 1494-1503 (1983).

31. Jones, L. H. *J. Chem. Phys.* **23**, 2105-2107 (1955).

32. Jin, G. B.; Skanthakumar, S.; Soderholm, L. *Inorg. Chem.* **51**, 3220-3230 (2012).

33. Almond, P. M.; Skanthakumar, S.; Soderholm, L.; Burns, P. C. *Chem. Mater.* **19**, 280-285 (2007).

34. Ikeda-Ohno, A.; Hennig, C.; Rossberg, A.; Funke, H.; Scheinost, A. C.; Bernhard, G.; Yaita, T. *Inorg. Chem.* **47**, 8294-8305 (2008).

35. Soderholm, L.; Antonio, M. R.; Williams, C.; Wasserman, S. R. *Anal. Chem.* **71**, 4622-4628 (1999).

36. Ikeda-Ohno, A.; Tsushima, S.; Takao, K.; Rossberg, A.; Funke, H.; Scheinost, A. C.; Bernhard, G.; Yaita, T.; Hennig, C. *Inorg. Chem.* **48**, 11779-11787 (2009).

37. Konings, R. J. M.; Morss, L. R.; Fuger, J. Thermodynamic Properties of Actinides and Actinide Compounds. In *The Chemistry of the Actinide and Transactinide Elements*, Morss, L. R.; Edelstein, N. M.;Fuger, J., Eds. Springer: The Netherlands; Vol. 4, pp 2113-2224 (2006).

38. Guillaumont, R.; Fanghanel, T.; Neck, V.; Fuger, J.; Palmer, D. A.; Grenthe, I.; Rand, M. H. *Update on the chemical thermodynamics of uranium, neptunium, plutonium, americium and technetium*. Elsevier: New York, NY; Vol. 5 (2003).

39. Neck, V.; Fanghänel, T.; Rudolph, G.; Kim, J. I. *Radiochim. Acta* **69**, 39-47 (1995).
